# Analysis of Fungal Microbiomes in Edible Medicinal Morindae Officinalis Radix and Alpiniae Oxyphyllae Fructus Using DNA Metabarcoding

**DOI:** 10.3390/foods11121748

**Published:** 2022-06-14

**Authors:** Wenjun Jiang, Xuyu Chen, Mengyue Guo, Jingsheng Yu, Meihua Yang, Xiaohui Pang

**Affiliations:** 1 Institute of Medicinal Plant Development, Chinese Academy of Medical Sciences & Peking Union Medical College, Beijing 100193, China; wenjunjiang0927@gmail.com (W.J.); guomy0908@hotmail.com (M.G.); yujsimplad@hotmail.com (J.Y.); mhyang@implad.ac.cn (M.Y.); 2 Hainan Provincial Key Laboratory of Resources Conservation and Development of Southern Medicine, Hainan Branch of the Institute of Medicinal Plant Development, Chinese Academy of Medical Sciences & Peking Union Medical College, Haikou 570311, China; chenxuyu-11@163.com

**Keywords:** Morindae Officinalis Radix, Alpiniae Oxyphyllae Fructus, DNA metabarcoding, fungal microbiome, toxigenic fungi

## Abstract

Morindae Officinalis Radix (MOR) and Alpiniae Oxyphyllae Fructus (AOF) have been widely used as dietary supplements and traditional herbal medicines for centuries. Fungal and mycotoxin contamination in MOR and AOF has been reported recently. In this study, fungi in MOR and AOF are first investigated using DNA metabarcoding, and the differences in fungal microbiome between moldy and non−moldy samples are analyzed. The results show that Ascomycota is the most prevailing fungus at the phylum level in MOR and AOF with relative abundances of 49.53–94.32% and 14.81–81.85%, respectively. *Penicillium* (1.86–76.14%), *Cladosporium* (1.82–56.65%), and *Trichoderma* (0.12–19.71%) are the dominant genera in MOR. *Penicillium* (0.27–56.06%), *Papiliotrema* (0.04–51.71%), and *Cladosporium* (3.08–44.41%) are the dominant genera in AOF. Two potential toxigenic fungi were detected, namely, *Trichoderma atroviride* and *Fusarium equiseti*. Moreover, the differences in fungal communities between moldy and non−moldy samples were monitored. In conclusion, DNA metabarcoding can be used to assess the fungal microbiome in edible medicinal herbs, thereby providing a basis for ensuring food safety and drug efficacy.

## 1. Introduction

Chinese herbal medicines (CHMs), which are widely used for clinical treatment and daily healthcare, have undoubtedly played a significant role in medical and healthcare industries. About 70–80% of the global population depends on herbal medicinal products in their primary healthcare [1]. The global market of medicinal plants is growing and expected to reach USD 5 trillion by 2050 [2]. The popularity of herbal products is increasing as they are considered natural or harmless [2]. Unfortunately, fungal contamination in CHMs is a concern. Since the sources of CHMs are diversified and the planting and processing are decentralized, CHMs may be contaminated by fungi at any link of the complex traditional Chinese medicine industry chain if good manufacturing procedures are not followed, and even mycotoxins may be produced by potential toxigenic fungi under suitable conditions. Cases of fungal and mycotoxin contamination associated with CHMs have been constantly reported worldwide and have attracted considerable global attention. A study on microbial contamination in 132 herbal medicines and 18 water samples used in the preparation of herbal medicines from northern Brazil showed that 31% of tested samples exceeded the safety limits for fungal growth [3]. Keter et al. assessed the risk of fungi in 100 herbal products from the Kenyan market, and the results revealed that 69% of the samples did not meet the requirements about microbial limits shown in Pharmacopoeia. Among these, *Aspergillus* and *Penicillium* were dominant [4]. In Latvia, Reinholds et al. investigated the contamination profile of multi−mycotoxin and fungi in 140 *Camellia sinensis* and 26 herbal teas; 87% of the tea samples were positive for fungi and 42% had 1 to 16 mycotoxins, for instance, ochratoxin A (OTA), deoxynivalenol, and aflatoxins (AFs) [5]. A Poland investigation on the occurrence of OTA and fumonisins in 79 herbs and spices showed that 31% of the samples were positive for fumonisins and 49% for OTA [6]. Another investigation on the fungal and multi−mycotoxin contamination in 48 root herbs marketed in China determined that all samples were affected by fungal contamination; 37.5% were positive for AFs and 16.67% for OTA [7]. Moldy CHMs affect drug efficacy, pose threats to consumer health, affect the export trade, and cause economic losses.

Morindae Officinalis Radix (MOR, Bajitian in Chinese), namely, the dried root of *Morinda officinalis* How. (Rubiaceae), has long been used as a tonic herbal medicine for tonifying the kidney, strengthening sinew and bone, and dispelling wind−dampness [8]. MOR is also a popular dietary supplement for daily healthcare (e.g., bone protection and gynecological and andrological healthcare) [9]. The constituents extracted from MOR, e.g., anthraquinones, oligosaccharides, polysaccharides, and iridoid glycosides, have various bioactive activities, including anti-osteoporosis, anti-depressant, pro-fertility, immune-regulatory, anti-inflammation, and antioxidant effects [10,11,12,13,14]. Alpiniae Oxyphyllae Fructus (AOF, Yizhi in Chinese), a famed edible medicinal herb, is from the dried, ripe fruit of *Alpinia oxyphylla* Miq. AOF has the functions of warming the kidney to secure essence to reduce urination, and warming the spleen to check diarrhea and constrain spittle [8]. Contemporary research shows the presence of sesquiterpenes, polysaccharides, diarylheptanoids, flavonoids, and volatile oils in AOF [15]. AOF and its extracts exhibit neuroprotective, anti-ulcer, anti-inflammatory, and anti-hyperuricemic effects, and have been used for the treatment of dementia, ulceration, and tumors [16,17,18]. The consumption and demand for MOR and AOF are high, owing to their outstanding pharmaceutical properties and edible values. MOR and AOF are mainly produced in Hainan, Guangdong, and Guangxi provinces, as well as in other tropical and subtropical regions of China [19], in which the climate situation contributes to the development of fungi and the production of mycotoxins [20]. MOR and AOF are easily affected by the contamination of fungi without obeying proper harvest, processing, transportation, and storage procedures. Therefore, it is necessary to comprehensively and efficiently investigate the fungal contamination before the use of MOR and AOF. Contamination of fungi is challenging to identify because of their complex morphological and taxonomic characteristics. Thus, a method that simultaneously and effectively analyzes fungal microbiomes in MOR and AOF is urgently desired.

DNA metabarcoding, an emerging culture-independent technique, refers to high-throughput multispecies (or higher-level taxon) identification using the total and typically degraded DNA extracted from an environmental sample (i.e., soil, water, and feces) [21]. It has a wide range of applications in the study of fungal ecology and provides new insights into fungal microbiomes in different environments. The internal transcribed spacer (ITS) region of rDNA was recommended as a universal DNA barcode marker for fungi [22]. Most amplicon sequencing studies of fungal diversity have focused on ITS1 or ITS2 sublocus [23]. In this study, we first use DNA metabarcoding to characterize the fungal microbiomes in MOR and AOF, and compare the differences between moldy and non−moldy samples.

## 2. Materials and Methods

### 2.1. Sampling

A total of 18 samples, including MOR and AOF, were collected from herbal markets in Hainan, Guangdong, and Guangxi. The samples were divided into two groups, according to the species (i.e., MOR and AOF), and four groups based on the presence or absence of macroscopic molds (i.e., MM and MA are the moldy MOR and AOF samples, respectively; NM and NA are the non-moldy MOR and AOF samples, respectively). The details of all samples are shown in Table 1.

### 2.2. DNA Extraction

About 3 g of MOR (the dried root) or AOF (the dried ripe fruit) samples was transferred into a 50 mL sterilized centrifuge tube and mixed with 20 mL of sterilized water, and shaken for 20 min by a vortex mixer. Then, the mixture was filtered by Millipore filter membrane (50 mm diameter, 0.2 μm pore size). Total DNA was extracted by the cetyltrimethyl ammonium bromide method [24].

### 2.3. Polymerase Chain Reaction (PCR) Amplification and High-Throughput Sequencing (HTS)

The ITS1 region of the fungi was amplified with the primer pairs ITS1F (5′-CTTGGTCATTTAGAGGAAGTAA-3′) [25] and ITS2R (5′-GCTGCGTTCTTCATCG ATGC-3′) [26]. PCR was performed on initial denaturation at 95 °C for 5 min, 34 cycles of denaturation at 95 °C for 45 s, 50 s annealing at 58 °C, 60 s elongation at 72 °C, and 10 min extension at 72 °C. The PCR product was detected by 2% agarose gel electrophoresis and purified using the Universal DNA Purification Kit (DP214) (TIANGEN Biotech Co., Ltd., Beijing, China). Then, the amplicons were sequenced on the IonS5^TM^XL platform (Thermofisher, Waltham, MA, USA). Raw reads were submitted to the National Center for Biotechnology Information Sequence Read Archive database under the accession numbers SAMN19591296–SAMN19591313.

### 2.4. Data Analysis

Low-quality regions of the sequences were removed by Cutadapt (version 1.9.1) [27], and then the barcode and primer sequences were cut off to obtain the raw reads. Chimeric sequences were removed using USEARCH (version 8.1.1861) [28], and clean reads were clustered into operational taxonomic units (OTUs) at a 97% similarity level by UPARSE (version 7.1) [29]. OTUs were annotated according to the UNITE database ranging from kingdom to species level [30] and verified via manual search. Five metrics, Shannon, Chao 1, ACE, Simpson, and Good’s coverage, were calculated by QIIME (version 1.9.1) [31] to assess alpha diversity. Statistical differences between MOR and AOF were examined by analysis of similarity (ANOSIM). Principal coordinate analysis (PCoA) on the basis of the Bray–Curtis distance matrix was applied to estimate the difference in the fungal community of samples from different species. The linear discriminant analysis effect size (LEfSe) algorithm (LDA score = 4.0) was performed to distinguish the differentially abundant taxa between two groups [32]. Samples were hierarchically clustered by the unweighted pair group method with arithmetic mean (UPGMA) based on unweighted UniFrac distances. R tools (version 2.15.3) were applied to plot the rarefaction curves, heat map, and Venn diagram.

## 3. Results

### 3.1. Diversity Analysis of Fungal Microbiomes in MOR and AOF Samples

**Figure 1:**
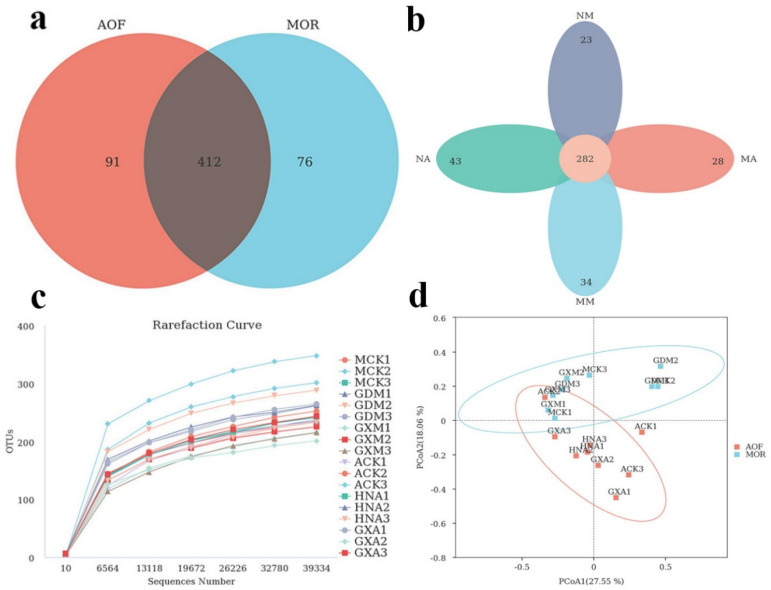
Analysis of fungal diversity in MOR and AOF samples. (**a**) Venn diagram of OTUs in the MOR and AOF groups; (**b**) Venn diagram of OTUs in the moldy and non-moldy samples; (**c**) Rarefaction curves for OTUs in all samples; and (**d**) PCoA diagram. of fungal compositions in samples.

A total of 1,413,703 valid ITS1 sequences with an average length of 235 bp were obtained from 18 samples. The sequences were clustered into 579 OTUs (≥97% similarity, Table S1). Venn analysis exhibited that 91 and 76 OTUs were, respectively, unique for AOF and MOR groups, and the remaining 412 OTUs were common in the two groups (Figure 1a). Moreover, 282 OTUs were shared by 4 groups, and 23, 34, 43, and 28 OTUs were, respectively, unique for NM, MM, NA, and MA groups (Figure 1b). With the number of sequences sampled increasing, the rarefaction curves of all samples were parallel to the *x*-axis, which indicates the reliability of the sequencing depth employed (Figure 1c). The 5 alpha-diversity indices were employed to analyze the richness, diversity, and coverage of fungal microbiomes in 18 samples (Table 2). The Good’s coverage was over 99.8%, which shows that the sampling depth satisfies the analysis requirements. In the MOR group, the ACE and Chao 1 indices of GDM were the highest, and the community richness was the highest. In contrast, GXM had the lowest community richness. The community diversity of GDM samples was the highest with the highest Shannon and Simpson indices. GXM had the lowest community diversity. Similarly, HNA and ACK in the AOF group had the highest community diversity and community richness, respectively. PCoA analysis showed that AOF and MOR groups were distinguishable (Figure 1d).

### 3.2. Composition of Fungal Microbiomes in MOR and AOF Samples

**Figure 2:**
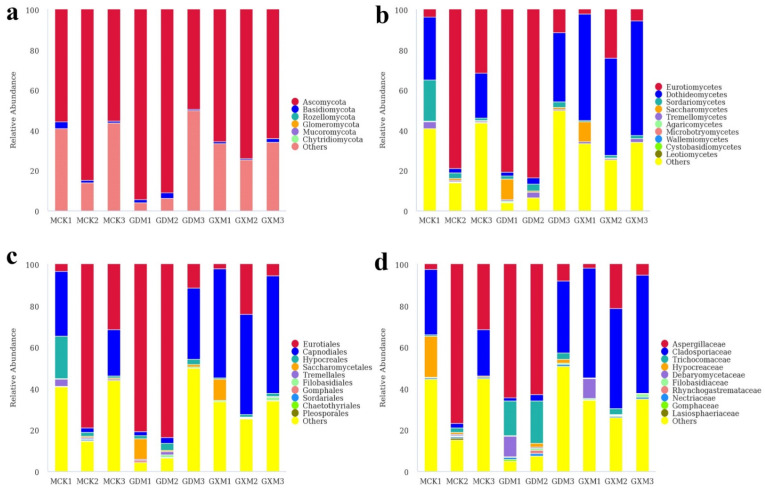
Fungal composition of the MOR samples at the phylum (**a**), class (**b**), order (**c**), and family (**d**) levels.

**Figure 3:**
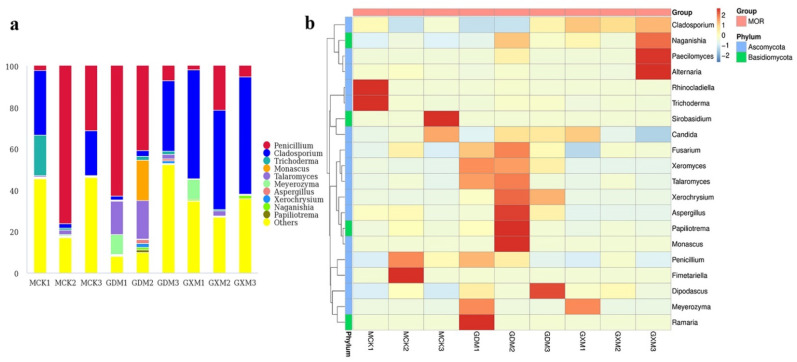
Composition analyses of the fungal microbiomes in the MOR samples. (**a**) Fungal composition in MOR samples at genus level; (**b**) Heatmap of the top 20 genera in MOR samples.

In the MOR group, the dominant phylum was Ascomycota with a relative abundance of 49.53–94.32%, whereas the other phyla were low in abundance (Figure 2a). Eurotiomycetes, Dothideomycetes, and Sordariomycetes were dominant at the class level, accounting for 2.09–78.91%, 1.88–57.02%, and 0.60–20.37% of the fungal reads, respectively (Figure 2b). At the order level, Capnodiales was predominant in MCK1, GDM3, GXM1, GXM2, and GXM3, whereas Eurotiales was predominant in MCK2, MCK3, GDM1, and GDM2 (Figure 2c). Further taxonomical classification demonstrated that Aspergillaceae (1.90–76.73%) was the most dominant at the family level, followed by Cladosporiaceae (1.83–56.78%) and Trichocomaceae (0.12–20.45%, Figure 2d). At the genus level, *Penicillium* (1.86–76.14%), *Cladosporium* (1.82–56.65%), *Trichoderma* (0.12–19.71%), *Monascus* (0.00–19.35%), and *Talaromyces* (0.18–19.71%) were the top 5 genera with the highest relative abundance (Figure 3a). The top 20 abundant genera were visualized using a heatmap, which showed the relative abundance of fungal genera in different samples (Figure 3b).

**Figure 4:**
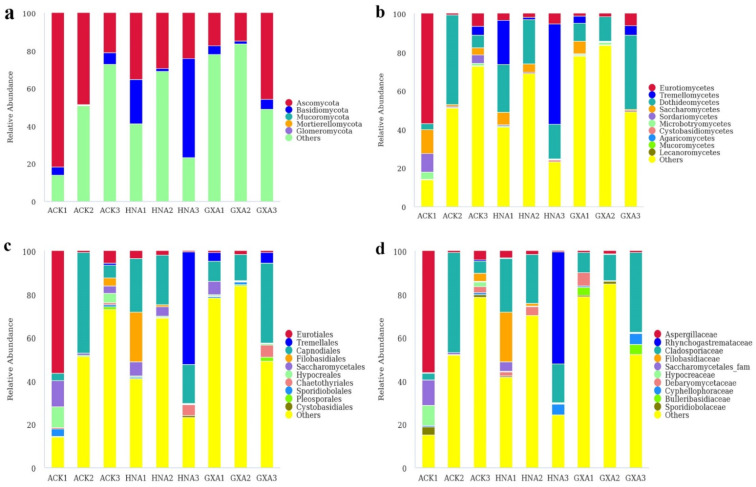
Fungal composition of the AOF samples at the phylum (**a**), class (**b**), order (**c**), and family (**d**) levels.

**Figure 5:**
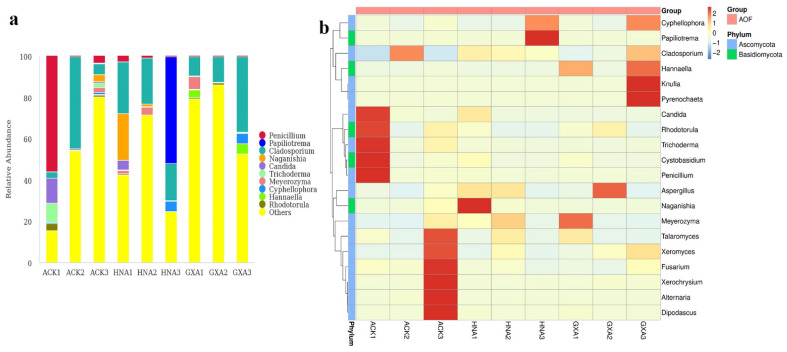
Composition analyses of the fungal microbiomes in the AOF samples. (**a**) Fungal composition in AOF samples at genus level; (**b**) Heatmap of the top 20 genera in AOF samples.

In the AOF group, Ascomycota was the most abundant phylum representing 14.81–81.85%, followed by Basidiomycota (0.36–52.57%, Figure 4a). At the class level, Eurotiomycetes (0.57–56.84%), Tremellomycetes (0.11–51.81%), and Dothideomycetes (3.13–46.37%) were prevalent (Figure 4b). Among the 39 orders detected, Eurotiales, Tremellales, and Capnodiales were dominant with the relative abundances of 0.41–56.29%, 0.09–51.78%, and 3.11–46.30%, respectively (Figure 4c). At the family level, the dominant fungi in samples ACK1 and HNA3 were Aspergillaceae (56.17%) and Rhynchogastremataceae (51.71%), respectively. Cladosporiaceae was dominant in the rest of the samples representing 5.71–46.30% (Figure 4d). *Penicillium* (0.27–56.06%), *Papiliotrema* (0.04–51.71%), and *Cladosporium* (3.08–44.41%) were dominant at the genus level (Figure 5a). *Candida* was the subdominant genus in the ACK1 with the relative abundance of 11.95%, while it accounted for low levels in other samples (0.00–4.51%). A heatmap of the 20 most abundant genera is presented in Figure 5b.

It is worth noting that a potential toxigenic fungus, namely, *Trichoderma atroviride*, was detected in all samples. Furthermore, another potential toxigenic fungus (*Fusarium equiseti*) was detected in four non-moldy samples (MCK1, MCK2, ACK1, and ACK3). The species and relative abundance of harmful fungi in each sample are shown in Supplementary Table S2.

### 3.3. Comparison of Fungal Microbiomes in MOR and AOF

**Figure 6:**
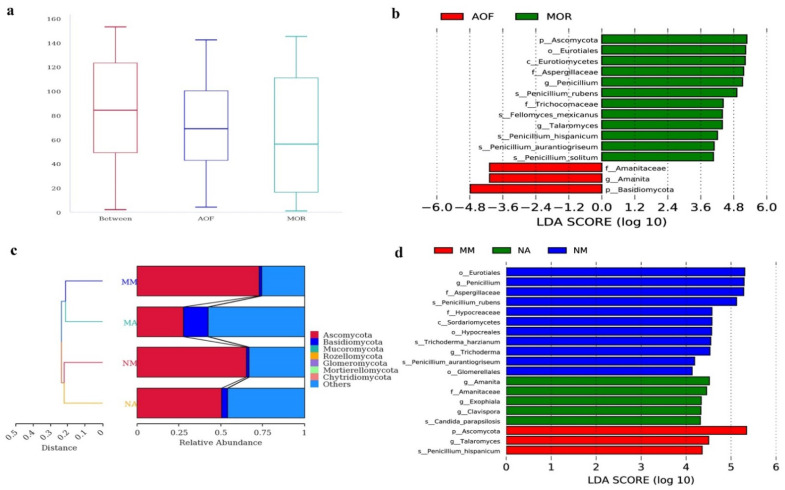
Comparison of fungal microbiomes in the MOR and AOF samples. (**a**) Evident difference in fungal microbiomes between the MOR and AOF groups based on ANOSIM; (**b**) Differentially abundant fungal taxa between the MOR and AOF groups; (**c**) UPGMA clustering based on unweighted UniFrac distance analysis; and (**d**) Differentially abundant fungal taxa between the moldy and non–moldy samples.

ANOSIM analysis was applied to test the difference between the two groups; the results show that MOR and AOF express statistical differences in fungal microbiomes (R = 0.2, *p* = 0.009, Figure 6a). LEfSe analysis was performed to calculate the differences in fungal taxa from the phylum to species level between different groups (LDA score = 4.0). In contrast to AOF samples, MOR samples had more abundant Ascomycota, but had less Basidiomycota. The relative abundance of the Aspergillaceae family was much higher in MOR samples, whereas Amanitaceae was more abundant in AOF samples. *Penicillium* and *Talaromyces* were detected more frequently in MOR samples, whereas *Amanita* was more abundant in AOF samples (Figure 6b).

As for the moldy and non-moldy samples, the result of hierarchical clustering analysis indicates that samples are clustered according to the presence or absence of macroscopic molds (Figure 6c). LEfSe analysis showed that none of the fungal taxa was found to be enriched in the MA group. In the NM group, three orders (Eurotiales, Hypocreales, and Glomerellales), two families (Aspergillaceae and Hypocreaceae), and two genera (*Penicillium* and *Trichoderma*) were enriched. In the NA group, one family (Amanitaceae) and three genera (*Amanita*, *Exophiala*, and *Clavispora*) were enriched. Moreover, *Talaromyces* was enriched in the MM group (Figure 6d).

## 4. Discussion

### 4.1. The Necessity of Characterizing Fungal Microbiomes in MOR and AOF Samples

MOR and AOF are derived from the roots and fruits of plants, respectively. They are susceptible to fungal contamination during pre- and post-harvest processes owing to the lack of standard management. In our study, all 18 samples were contaminated with fungi. The relative abundances of fungi were varied in different samples. Ascomycota, Eurotiomycetes, Eurotiales, and Aspergillaceae were generally dominant at the phylum, class, order, and family levels in AOF and MOR samples, respectively. In general, *Penicillium* and *Cladosporium* were the dominant genera. In our previous studies, the predominant genus in four seed herbs (Platycladi Semen, Myristicae Semen, and Cassiae Semen) was *Aspergillus* [33,34,35]. The relationship between dominant fungi and herbs has not been well explained, owing to the complex factors that influence the fungal community. The storage conditions and matrix composition of CHMs may be responsible for the difference in fungal microbiomes between MOR and AOF. Moreover, the fungal microbiomes between moldy and non-moldy samples are different. All samples were clustered based on the presence or absence of visible molds in UPGMA, thus indicating the meaningfulness of grouping. *Cladosporium* and *Penicillium* were dominant in moldy and non-moldy samples, respectively.

Notably, all samples were contaminated with a potential toxigenic fungus, namely, *Trichoderma atroviride*. Additionally, four non-moldy samples were contaminated with another toxigenic fungus (*Fusarium equiseti*). Trichothecenes and zearalenone, which disturb hormonal balance and cause numerous diseases in the reproductive system, can be produced by *F. equiseti* [36,37]. The findings of our study are consistent with another study; potential mycotoxin-producing fungi were detected in non-moldy and moldy Ziziphi Spinosae Semen samples [38]. Similarly, Wei et al. detected AFs and OTA in six moldy and nine non-visible moldy *Glycyrrhiza uralensis* samples collected from China [39]. Therefore, the safety assessment of CHMs should not depend on whether macroscopic molds are present or not. Characterizing fungal microbiomes, especially potential toxigenic fungi, in MOR and AOF samples is essential, which is providing an early warning for mycotoxin contamination.

### 4.2. DNA Metabarcoding Is a Powerful Tool for the Analysis of Fungal Diversity in CHMs

Mycotoxins, the toxic secondary metabolites, can lead to numerous health problems and even death in just small intakes, and they are mainly produced by *Penicillium*, *Aspergillus*, and *Fusarium* [40]. Mycotoxins cannot degrade during storage and are difficult to remove from CHMs because of their thermostability [41,42]. Moreover, considerable transfer rates of AFs and OTA have been observed from herbal medicines to decoctions and pose a threat to consumer health [43,44]. Comprehensive and efficient fungi identification in CHMs is an important basis for preventing fungal and mycotoxin contamination. Current assessment methods for fungal contamination in CHMs are based on fungal isolation and culture, which may affect the real extent of fungal diversities because of the difficulty to cultivate and isolate certain microorganisms. The application of DNA metabarcoding could overcome this limitation and better reveal the fungal diversity. For example, Xia et al. found that 55 genera in Chinese Cordyceps that were not observed by culture-dependent methods were identified through Illumina Miseq sequencing [45]. Similarly, another study indicated that the number of fungi in Coix Seed detected by HTS was considerably greater than that by potato dextrose agar medium [46]. In addition, DNA metabarcoding also overcomes some of the limitations of a culture-dependent approach (e.g., time-consuming and complicated procedure). It could be used as an effective tool for the simultaneous characterization of numerous microbial species. In previous studies, DNA metabarcoding has been successfully applied in the investigation of fungal contamination in Platycladi Semen [33], Myristicae Semen [34], and Cassiae Semen [35]. In the present study, DNA metabarcoding efficiently analyzed the fungal microbiomes in MOR and AOF. Thus, it can serve as a powerful tool for identifying fungi in CHMs.

## 5. Conclusions

The fungal microbiomes in MOR and AOF were first investigated by DNA metabarcoding. The results highlight the effectiveness of the technique to analyze fungal diversity in CHMs and the need for the surveillance of marketed herbs to guarantee quality. This study provided an early warning for subsequent potential mycotoxin biosynthesis and can serve as a basis for the safe use of edible medicinal herbs.

## Figures and Tables

**Table 1:** Voucher information and GenBank accession numbers of the samples.

Name	Sample ID	Group	Moldy	Group	Source	GenBank Accession No.
Morindae Officinalis Radix	MCK1	MOR	No	NM	Haikou, Hainan	SAMN19591296
Morindae Officinalis Radix	MCK2	MOR	No	NM	Haikou, Hainan	SAMN19591297
Morindae Officinalis Radix	MCK3	MOR	No	NM	Haikou, Hainan	SAMN19591298
Morindae Officinalis Radix	GDM1	MOR	Yes	MM	Qingping, Guangdong	SAMN19591299
Morindae Officinalis Radix	GDM2	MOR	Yes	MM	Qingping, Guangdong	SAMN19591300
Morindae Officinalis Radix	GDM3	MOR	Yes	MM	Qingping, Guangdong	SAMN19591301
Morindae Officinalis Radix	GXM1	MOR	Yes	MM	Yulin, Guangxi	SAMN19591302
Morindae Officinalis Radix	GXM2	MOR	Yes	MM	Yulin, Guangxi	SAMN19591303
Morindae Officinalis Radix	GXM3	MOR	Yes	MM	Yulin, Guangxi	SAMN19591304
Alpiniae Oxyphyllae Fructus	ACK1	AOF	No	NA	Haikou, Hainan	SAMN19591305
Alpiniae Oxyphyllae Fructus	ACK2	AOF	No	NA	Haikou, Hainan	SAMN19591306
Alpiniae Oxyphyllae Fructus	ACK3	AOF	No	NA	Haikou, Hainan	SAMN19591307
Alpiniae Oxyphyllae Fructus	HNA1	AOF	Yes	MA	Qingping, Guangdong	SAMN19591308
Alpiniae Oxyphyllae Fructus	HNA2	AOF	Yes	MA	Qingping, Guangdong	SAMN19591309
Alpiniae Oxyphyllae Fructus	HNA3	AOF	Yes	MA	Qingping, Guangdong	SAMN19591310
Alpiniae Oxyphyllae Fructus	GXA1	AOF	Yes	MA	Yulin, Guangxi	SAMN19591311
Alpiniae Oxyphyllae Fructus	GXA2	AOF	Yes	MA	Yulin, Guangxi	SAMN19591312
Alpiniae Oxyphyllae Fructus	GXA3	AOF	Yes	MA	Yulin, Guangxi	SAMN19591313

**Table 2:** Alpha-diversity indices of samples.

Sample No.	Shannon	Simpson	Chao1	Ace	Goods_Coverage
MCK1	4.055	0.857	249.794	257.651	0.999
MCK2	3.791	0.779	364.441	354.497	0.998
MCK3	3.971	0.865	269.150	282.910	0.999
GDM1	4.412	0.910	315.250	297.879	0.998
GDM2	4.618	0.902	321.588	323.193	0.999
GDM3	4.068	0.840	347.370	333.659	0.998
GXM1	3.269	0.721	274.000	280.869	0.998
GXM2	3.441	0.751	243.077	253.748	0.999
GXM3	3.098	0.679	280.278	294.317	0.998
ACK1	3.122	0.740	349.241	330.054	0.998
ACK2	3.403	0.766	290.250	300.323	0.998
ACK3	4.050	0.745	424.241	402.027	0.998
HNA1	3.706	0.843	294.286	309.556	0.998
HNA2	4.886	0.931	315.038	308.207	0.999
HNA3	3.786	0.803	273.933	276.385	0.999
GXA1	3.476	0.712	311.838	322.078	0.998
GXA2	3.398	0.761	238.037	244.68	0.999
GXA3	4.064	0.839	257.500	269.869	0.999

## Data Availability

All related data and methods are presented in this paper. Additional inquiries should be addressed to the corresponding author.

## Supplementary Materials

The following supporting information can be downloaded at: https://www.mdpi.com/article/10.3390/foods11121748/s1, Table S1: Taxonomical classification of OTUs; Table S2: The species and relative abundance of potential toxigenic fungi in each sample.
