# Supplementary material for: Analysis of Fungal Microbiomes in Edible Medicinal Morindae Officinalis Radix and Alpiniae Oxyphyllae Fructus Using DNA Metabarcoding
Source: Foods. 2022 Jun 14;11(12):1748. doi: 10.3390/foods11121748 (PMC9222558; doi:10.3390/foods11121748)
Supplement: Supplementary file 1 [file foods-11-01748-s001.zip › foods-1748356-supplementary.pdf]

foods

MDPI

Supplementary material

Analysis of fungal microbiome in edible-medicinal Morindae Officinalis Radix and Alpiniae Oxyphyllae Fructus using DNA metabarcoding

Wenjun Jiang[1,†], Xuyu Chen[2,†], Mengyue Guo[1], Jingsheng Yu[1], Meihua Yang[1] and Xiaohui Pang [1,*]

1 Institute of Medicinal Plant Development, Chinese Academy of Medical Sciences & Peking Union Medical College, Beijing 100193, China; wenjunjiang0927@gmail.com (W. J.), guomy0908@hotmail.com (M. G.), yujsIMPLAD@hotmail.com (J.Y.), mhyang@implad.ac.cn (M. Y.)
2 Hainan Provincial Key Laboratory of Resources Conservation and Development of Southern Medicine, Hainan Branch of the Institute of Medicinal Plant Development, Chinese Academy of Medical Sciences & Peking Union Medical College, Haikou 570311, China; chenxuyu-11@163.com (X. C.)
* Correspondence: xhpang@implad.ac.cn
† These authors contributed equally to this work.

Table S1. Taxonomical classification of OTUs.

ID	taxonomy
OTU_1	k__Fungi;p__Ascomycota;c__Dothideomycetes;o__Capnodiales;f__Cladosporiaceae;g__Cladosporium;s__
OTU_2	k__Fungi;p__;c__;o__;f__;g__;s__
OTU_3	k__Fungi;p__Ascomycota;c__Eurotiomycetes;o__Eurotiales;f__Aspergillaceae;g__Penicillium;s__
OTU_4	k__Fungi;p__;c__;o__;f__;g__;s__
OTU_5	k__Fungi;p__Ascomycota;c__Eurotiomycetes;o__Eurotiales;f__Aspergillaceae;g__Penicillium;s__
OTU_6	k__Fungi;p__Basidiomycota;c__Tremellomycetes;o__Tremellales;f__Rhynchogastremataceae;g__Papiliotrema;s__Papiliotrema_aurea
OTU_7	k__Fungi;p__Basidiomycota;c__Tremellomycetes;o__Filobasidiales;f__Filobasidiaceae;g__Naganishia;s__
OTU_8	k__Fungi;p__Ascomycota;c__Sordariomycetes;o__Hypocreales;f__Hypocreaceae;g__Trichoderma;s__
OTU_9	k__Fungi;p__Ascomycota;c__Sordariomycetes;o__Hypocreales;f__Hypocreaceae;g__Trichoderma;s__Trichoderma_
OTU_10	k__Fungi;p__Ascomycota;c__Eurotiomycetes;o__Eurotiales;f__Aspergillaceae;g__Monascus;s__Monascus_sanguineus
OTU_11	k__Fungi;p__Ascomycota;c__Eurotiomycetes;o__Eurotiales;f__Aspergillaceae;g__Penicillium;s__Penicillium_hispanicum
OTU_12	k__Fungi;p__Basidiomycota;c__Tremellomycetes;o__Tremellales;f__Bulleribasidiaceae;g__Hannaella;s__Hannaella_oryzae
OTU_13	k__Fungi;p__Ascomycota;c__Saccharomycetes;o__Saccharomycetales;f__Saccharomycetales_fam_Incertae_sedis;g__Candida;s__Candida_parapsilosis
OTU_14	k__Fungi;p__Ascomycota;c__Eurotiomycetes;o__Eurotiales;f__Aspergillaceae;g__Penicillium;s__
OTU_15	k__Fungi;p__Ascomycota;c__Eurotiomycetes;o__Eurotiales;f__Trichocomaceae;g__Talaromyces;s__
OTU_16	k__Fungi;p__Ascomycota;c__Eurotiomycetes;o__Chaetothyriales;f__Cyphellophoraceae;g__Cyphellophora;s__Cyphellophora_europaea
OTU_17	k__Fungi;p__Ascomycota;c__Saccharomycetes;o__Saccharomycetales;f__Saccharomycetales_fam_Incertae_sedis;g__Candida;s__Candida_tropicalis
OTU_18	k__Fungi;p__Basidiomycota;c__Microbotryomycetes;o__Sporidiobolales;f__Sporidiobolaceae;g__Rhodotorula;s__
OTU_19	k__Fungi;p__Ascomycota;c__Sordariomycetes;o__Hypocreales;f__Nectriaceae;g__Fusarium;s__
OTU_20	k__Fungi;p__Basidiomycota;c__Tremellomycetes;o__Tremellales;f__;g__;s__
OTU_21	k__Fungi;p__;c__;o__;f__;g__;s__
OTU_22	k__Fungi;p__Ascomycota;c__Eurotiomycetes;o__Eurotiales;f__Aspergillaceae;g__Penicillium;s__
OTU_23	k__Fungi;p__Ascomycota;c__Sordariomycetes;o__Hypocreales;f__Nectriaceae;g__Fusarium;s__
OTU_24	k__Fungi;p__Ascomycota;c__Eurotiomycetes;o__Eurotiales;f__Trichocomaceae;g__Xerochrysium;s__
OTU_25	k__Fungi;p__Ascomycota;c__Dothideomycetes;o__Pleosporales;f__Cucurbitariaceae;g__Pyrenochaeta;s__Pyrenochaeta_unguis-hominis

OTU_26	k__Fungi;p__Ascomycota;c__Eurotiomycetes;o__Eurotiales;f__Aspergillaceae;g__Xeromyces;s__Xeromyces_bisporus
OTU_27	k__Fungi;p__Basidiomycota;c__Tremellomycetes;o__Tremellales;f__Rhynchogastremataceae;g__Papiliotrema;s__Papiliotrema_flavescens
OTU_28	k__Fungi;p__;c__;o__;f__;g__;s__
OTU_29	k__Fungi;p__Ascomycota;c__Sordariomycetes;o__Hypocreales;f__Nectriaceae;g__Volutella;s__
OTU_30	k__Fungi;p__Basidiomycota;c__Microbotryomycetes;o__Sporidiobolales;f__Sporidiobolaceae;g__Rhodotorula;s__Rhodotorula_mucilaginosa
OTU_31	k__Fungi;p__;c__;o__;f__;g__;s__
OTU_32	k__Fungi;p__Ascomycota;c__Eurotiomycetes;o__Eurotiales;f__Aspergillaceae;g__Aspergillus;s__
OTU_33	k__Fungi;p__Ascomycota;c__Eurotiomycetes;o__Eurotiales;f__Trichocomaceae;g__Talaromyces;s__
OTU_34	k__Fungi;p__;c__;o__;f__;g__;s__
OTU_35	k__Fungi;p__Ascomycota;c__Dothideomycetes;o__Capnodiales;f__Cladosporiaceae;g__Cladosporium;s__
OTU_36	k__Fungi;p__Ascomycota;c__Dothideomycetes;o__Pleosporales;f__Pleosporaceae;g__Alternaria;s__
OTU_37	k__Fungi;p__;c__;o__;f__;g__;s__
OTU_38	k__Fungi;p__Ascomycota;c__Eurotiomycetes;o__Eurotiales;f__Aspergillaceae;g__Penicillium;s__
OTU_39	k__Fungi;p__;c__;o__;f__;g__;s__
OTU_40	k__Fungi;p__;c__;o__;f__;g__;s__
OTU_41	k__Fungi;p__;c__;o__;f__;g__;s__
OTU_42	k__Fungi;p__Basidiomycota;c__Tremellomycetes;o__Tremellales;f__Tremellaceae;g__Cryptococcus;s__
OTU_43	k__Fungi;p__Ascomycota;c__Eurotiomycetes;o__Eurotiales;f__Aspergillaceae;g__Penicillium;s__
OTU_44	k__Fungi;p__Ascomycota;c__Dothideomycetes;o__Pleosporales;f__;g__;s__
OTU_45	k__Fungi;p__Ascomycota;c__Eurotiomycetes;o__Eurotiales;f__Aspergillaceae;g__Aspergillus;s__Aspergillus_versicolor
OTU_46	k__Fungi;p__Basidiomycota;c__Tremellomycetes;o__Tremellales;f__Sirobasidiaceae;g__Sirobasidium;s__
OTU_47	k__Fungi;p__Ascomycota;c__Eurotiomycetes;o__Eurotiales;f__Trichocomaceae;g__Talaromyces;s__
OTU_48	k__Fungi;p__;c__;o__;f__;g__;s__
OTU_49	k__Fungi;p__Ascomycota;c__Sordariomycetes;o__Sordariales;f__Lasiosphaeriaceae;g__Fimetariella;s__Fimetariella_rabenhorstii
OTU_50	k__Fungi;p__Ascomycota;c__Saccharomycetes;o__Saccharomycetales;f__Debaryomycetaceae;g__Meyerozyma;s__
OTU_51	k__Fungi;p__;c__;o__;f__;g__;s__
OTU_52	k__Fungi;p__;c__;o__;f__;g__;s__
OTU_53	k__Fungi;p__Basidiomycota;c__Tremellomycetes;o__Tremellales;f__Bulleribasidiaceae;g__Hannaella;s__
OTU_54	k__Fungi;p__;c__;o__;f__;g__;s__
OTU_55	k__Fungi;p__;c__;o__;f__;g__;s__
OTU_56	k__Fungi;p__Ascomycota;c__Saccharomycetes;o__Saccharomycetales;f__Debaryomycetaceae;g__Yamadazyma;s__
OTU_57	k__Fungi;p__;c__;o__;f__;g__;s__
OTU_58	k__Fungi;p__Basidiomycota;c__Cystobasidiomycetes;o__Cystobasidiales;f__Cystobasidiaceae;g__;s__
OTU_59	k__Fungi;p__Ascomycota;c__Dothideomycetes;o__Pleosporales;f__Phaeosphaeriaceae;g__;s__
OTU_60	k__Fungi;p__Basidiomycota;c__Tremellomycetes;o__Tremellales;f__Rhynchogastremataceae;g__Papiliotrema;s__
OTU_61	k__Fungi;p__Ascomycota;c__Dothideomycetes;o__Pleosporales;f__Phaeosphaeriaceae;g__Phaeosphaeria;s__
OTU_62	k__Fungi;p__Basidiomycota;c__Tremellomycetes;o__Filobasidiales;f__Filobasidiaceae;g__Naganishia;s__
OTU_63	No blast hit
OTU_64	k__Fungi;p__;c__;o__;f__;g__;s__
OTU_65	k__Fungi;p__;c__;o__;f__;g__;s__
OTU_66	k__Fungi;p__Ascomycota;c__;o__;f__;g__;s__
OTU_67	k__Fungi;p__;c__;o__;f__;g__;s__
OTU_68	k__Fungi;p__Basidiomycota;c__Tremellomycetes;o__Tremellales;f__Rhynchogastremataceae;g__Papiliotrema;s__Papiliotrema_laurentii
OTU_69	k__Fungi;p__;c__;o__;f__;g__;s__
OTU_70	k__Fungi;p__Ascomycota;c__Eurotiomycetes;o__Eurotiales;f__Aspergillaceae;g__Aspergillus;s__
OTU_71	k__Fungi;p__;c__;o__;f__;g__;s__
OTU_72	k__Fungi;p__Ascomycota;c__Saccharomycetes;o__Saccharomycetales;f__Debaryomycetaceae;g__Meyerozyma;s__
OTU_73	k__Fungi;p__Ascomycota;c__Eurotiomycetes;o__Eurotiales;f__Aspergillaceae;g__Penicillium;s__
OTU_74	k__Fungi;p__Basidiomycota;c__Tremellomycetes;o__Tremellales;f__Bulleraceae;g__Bullera;s__Bullera_penniseticola
OTU_75	k__Fungi;p__Basidiomycota;c__Agaricomycetes;o__Agaricales;f__Tricholomataceae;g__Tricholoma;s__Tricholoma_matsutake

OTU_76	k__Fungi;p__Ascomycota;c__Eurotiomycetes;o__Chaetothyriales;f__Herpotrichiellaceae;g__Rhinocladiella;s__
OTU_77	k__Fungi;p__;c__;o__;f__;g__;s__
OTU_78	k__Fungi;p__Ascomycota;c__Saccharomycetes;o__Saccharomycetales;f__;g__;s__
OTU_79	k__Fungi;p__Ascomycota;c__Eurotiomycetes;o__Eurotiales;f__Trichocomaceae;g__Talaromyces;s__
OTU_80	k__Fungi;p__Ascomycota;c__Saccharomycetes;o__Saccharomycetales;f__Saccharomycetales_fam_Incertae_sedis;g__ Candida;s__Candida_metapsilosis
OTU_81	k__Fungi;p__Ascomycota;c__Sordariomycetes;o__Microascales;f__Ceratocystidaceae;g__Ceratocystis;s__
OTU_82	k__Fungi;p__;c__;o__;f__;g__;s__
OTU_83	k__Fungi;p__Ascomycota;c__Eurotiomycetes;o__Eurotiales;f__Aspergillaceae;g__Penicillium;s__
OTU_84	k__Fungi;p__;c__;o__;f__;g__;s__
OTU_85	k__Fungi;p__Ascomycota;c__Sordariomycetes;o__Glomerellales;f__Glomerellaceae;g__Colletotrichum;s__
OTU_86	k__Fungi;p__Ascomycota;c__Eurotiomycetes;o__Eurotiales;f__Trichocomaceae;g__Talaromyces;s__
OTU_87	k__Fungi;p__Basidiomycota;c__Cystobasidiomycetes;o__Cystobasidiales;f__Cystobasidiaceae;g__Occultifur;s__Occu ltifur_externus
OTU_88	k__Fungi;p__Ascomycota;c__Eurotiomycetes;o__Chaetothyriales;f__Trichomeriaceae;g__Knufia;s__
OTU_89	k__Fungi;p__Ascomycota;c__Sordariomycetes;o__Sordariales;f__Cephalothecaceae;g__Phialemonium;s__
OTU_90	k__Fungi;p__Basidiomycota;c__Wallemiomycetes;o__Wallemiales;f__Wallemiaceae;g__Wallemia;s__Wallemia_melli cola
OTU_91	k__Fungi;p__;c__;o__;f__;g__;s__
OTU_92	k__Fungi;p__Ascomycota;c__Sordariomycetes;o__Hypocreales;f__Clavicipitaceae;g__Paecilomyces;s__
OTU_93	k__Fungi;p__Ascomycota;c__Eurotiomycetes;o__Eurotiales;f__Trichocomaceae;g__Talaromyces;s__
OTU_94	k__Fungi;p__Ascomycota;c__Saccharomycetes;o__Saccharomycetales;f__Saccharomycetales_fam_Incertae_sedis;g__ Candida;s__
OTU_95	k__Fungi;p__;c__;o__;f__;g__;s__
OTU_96	k__Fungi;p__;c__;o__;f__;g__;s__
OTU_97	k__Fungi;p__;c__;o__;f__;g__;s__
OTU_98	k__Fungi;p__;c__;o__;f__;g__;s__
OTU_99	k__Fungi;p__Ascomycota;c__Eurotiomycetes;o__Eurotiales;f__Aspergillaceae;g__Penicillium;s__
OTU_100	k__Fungi;p__Ascomycota;c__Sordariomycetes;o__Hypocreales;f__Nectriaceae;g__;s__
OTU_101	k__Fungi;p__Basidiomycota;c__Microbotryomycetes;o__Sporidiobolales;f__Sporidiobolaceae;g__Rhodotorula;s__
OTU_102	k__Fungi;p__;c__;o__;f__;g__;s__
OTU_103	k__Fungi;p__Ascomycota;c__Sordariomycetes;o__Hypocreales;f__Nectriaceae;g__Fusicolla;s__
OTU_104	k__Fungi;p__Basidiomycota;c__Tremellomycetes;o__Tremellales;f__Rhynchogastremataceae;g__Papiliotrema;s__
OTU_105	k__Fungi;p__;c__;o__;f__;g__;s__
OTU_106	k__Fungi;p__Ascomycota;c__Leotiomycetes;o__Helotiales;f__Vibrisseaceae;g__Phialocephala;s__Phialocephala_forti nii
OTU_107	k__Fungi;p__Ascomycota;c__Sordariomycetes;o__Hypocreales;f__Nectriaceae;g__Xenoacremonium;s__
OTU_108	k__Fungi;p__Ascomycota;c__Eurotiomycetes;o__Eurotiales;f__Trichocomaceae;g__Talaromyces;s__
OTU_109	k__Fungi;p__Ascomycota;c__Sordariomycetes;o__Glomerellales;f__Plectosphaerellaceae;g__Plectosphaerella;s__
OTU_110	k__Fungi;p__Basidiomycota;c__Cystobasidiomycetes;o__Cystobasidiomycetes_ord_Incertae_sedis;f__Symmetrospor aceae;g__Symmetrospora;s__
OTU_111	k__Fungi;p__Ascomycota;c__Eurotiomycetes;o__Eurotiales;f__Trichocomaceae;g__Talaromyces;s__
OTU_112	k__Fungi;p__Ascomycota;c__Eurotiomycetes;o__Eurotiales;f__Aspergillaceae;g__Penicillium;s__
OTU_113	k__Fungi;p__Ascomycota;c__Leotiomycetes;o__Rhytismatales;f__Cudoniaceae;g__Spathularia;s__
OTU_114	k__Fungi;p__Ascomycota;c__Saccharomycetes;o__Saccharomycetales;f__Dipodascaceae;g__Geotrichum;s__
OTU_115	k__Fungi;p__Ascomycota;c__Eurotiomycetes;o__Eurotiales;f__Aspergillaceae;g__;s__
OTU_116	k__Fungi;p__;c__;o__;f__;g__;s__
OTU_117	k__Fungi;p__Ascomycota;c__Eurotiomycetes;o__Eurotiales;f__Aspergillaceae;g__Aspergillus;s__
OTU_118	k__Fungi;p__Mucoromycota;c__Mucoromycetes;o__Mucorales;f__Mucoraceae;g__Apophysomyces;s__
OTU_119	k__Fungi;p__;c__;o__;f__;g__;s__
OTU_120	k__Fungi;p__;c__;o__;f__;g__;s__
OTU_121	k__Fungi;p__;c__;o__;f__;g__;s__
OTU_122	k__Fungi;p__Ascomycota;c__Sordariomycetes;o__Hypocreales;f__Cordycipitaceae;g__Beauveria;s__Beauveria_felin a
OTU_123	k__Fungi;p__;c__;o__;f__;g__;s__
OTU_124	k__Fungi;p__Basidiomycota;c__Tremellomycetes;o__Tremellales;f__;g__;s__
OTU_125	k__Fungi;p__;c__;o__;f__;g__;s__

OTU_126	k__Fungi;p__;c__;o__;f__;g__;s__
OTU_127	k__Fungi;p__Basidiomycota;c__Tremellomycetes;o__Filobasidiales;f__Filobasidiaceae;g__Naganishia;s__
OTU_128	k__Fungi;p__;c__;o__;f__;g__;s__
OTU_129	k__Fungi;p__;c__;o__;f__;g__;s__
OTU_130	k__Fungi;p__;c__;o__;f__;g__;s__
OTU_131	k__Fungi;p__;c__;o__;f__;g__;s__
OTU_132	k__Fungi;p__;c__;o__;f__;g__;s__
OTU_133	k__Fungi;p__;c__;o__;f__;g__;s__
OTU_134	k__Fungi;p__Basidiomycota;c__Tremellomycetes;o__Tremellales;f__Rhynchogastremataceae;g__Papiliotrema;s__
OTU_135	k__Fungi;p__Ascomycota;c__Eurotiomycetes;o__Eurotiales;f__Trichocomaceae;g__Talaromyces;s__
OTU_136	k__Fungi;p__Ascomycota;c__Saccharomycetes;o__Saccharomycetales;f__Dipodascaceae;g__Geotrichum;s__
OTU_137	k__Fungi;p__Ascomycota;c__Sordariomycetes;o__Hypocreales;f__;g__;s__
OTU_138	k__Fungi;p__;c__;o__;f__;g__;s__
OTU_139	k__Fungi;p__Ascomycota;c__Saccharomycetes;o__Saccharomycetales;f__Metschnikowiaceae;g__Kodamaea;s__Kodamaea_ohmeri
OTU_140	k__Fungi;p__Ascomycota;c__Sordariomycetes;o__Hypocreales;f__Nectriaceae;g__Fusarium;s__
OTU_141	k__Fungi;p__Ascomycota;c__Eurotiomycetes;o__Chaetothyriales;f__Cyphellophoraceae;g__Cyphellophora;s__Cyphellophora_oxyspora
OTU_142	k__Fungi;p__Ascomycota;c__Eurotiomycetes;o__Eurotiales;f__Aspergillaceae;g__Penicillium;s__
OTU_143	k__Fungi;p__Ascomycota;c__Saccharomycetes;o__Saccharomycetales;f__Dipodascaceae;g__;s__
OTU_144	No blast hit
OTU_145	k__Fungi;p__Ascomycota;c__Sordariomycetes;o__Hypocreales;f__Hypocreaceae;g__Trichoderma;s__Trichoderma_atroviride
OTU_146	k__Fungi;p__;c__;o__;f__;g__;s__
OTU_147	k__Fungi;p__Ascomycota;c__Eurotiomycetes;o__Eurotiales;f__Aspergillaceae;g__;s__
OTU_148	k__Fungi;p__Ascomycota;c__Eurotiomycetes;o__Eurotiales;f__Aspergillaceae;g__Penicillium;s__
OTU_149	k__Fungi;p__Ascomycota;c__;o__;f__;g__;s__
OTU_150	k__Fungi;p__Ascomycota;c__Saccharomycetes;o__Saccharomycetales;f__Dipodascaceae;g__Geotrichum;s__
OTU_151	k__Fungi;p__;c__;o__;f__;g__;s__
OTU_152	k__Fungi;p__;c__;o__;f__;g__;s__
OTU_153	k__Fungi;p__Basidiomycota;c__Geminibasidiomycetes;o__Geminibasidiales;f__Geminibasidiaceae;g__Geminibasidium;s__
OTU_154	k__Fungi;p__;c__;o__;f__;g__;s__
OTU_155	k__Fungi;p__Ascomycota;c__Eurotiomycetes;o__Eurotiales;f__Trichocomaceae;g__Xerochrysium;s__Xerochrysium_dermatitidis
OTU_156	k__Fungi;p__;c__;o__;f__;g__;s__
OTU_157	k__Fungi;p__;c__;o__;f__;g__;s__
OTU_158	k__Fungi;p__;c__;o__;f__;g__;s__
OTU_159	k__Fungi;p__;c__;o__;f__;g__;s__
OTU_160	k__Fungi;p__Ascomycota;c__Saccharomycetes;o__Saccharomycetales;f__Saccharomycetales_fam_Incertae_sedis;g__Candida;s__
OTU_161	k__Fungi;p__;c__;o__;f__;g__;s__
OTU_162	k__Fungi;p__;c__;o__;f__;g__;s__
OTU_163	k__Fungi;p__Ascomycota;c__Eurotiomycetes;o__Chaetothyriales;f__Cyphellophoraceae;g__Cyphellophora;s__
OTU_164	k__Fungi;p__;c__;o__;f__;g__;s__
OTU_165	k__Fungi;p__;c__;o__;f__;g__;s__
OTU_166	k__Fungi;p__;c__;o__;f__;g__;s__
OTU_167	k__Fungi;p__Ascomycota;c__Leotiomycetes;o__Helotiales;f__;g__;s__
OTU_168	k__Fungi;p__;c__;o__;f__;g__;s__
OTU_169	k__Fungi;p__Basidiomycota;c__Agaricomycetes;o__Russulales;f__Russulaceae;g__Russula;s__
OTU_170	k__Fungi;p__;c__;o__;f__;g__;s__
OTU_171	k__Fungi;p__Ascomycota;c__Saccharomycetes;o__Saccharomycetales;f__Saccharomycetales_fam_Incertae_sedis;g__Candida;s__Candida_parapsilosis
OTU_172	k__Fungi;p__Basidiomycota;c__Tremellomycetes;o__Tremellales;f__Rhynchogastremataceae;g__Papiliotrema;s__
OTU_173	k__Fungi;p__Ascomycota;c__Eurotiomycetes;o__Eurotiales;f__Trichocomaceae;g__Talaromyces;s__
OTU_174	k__Fungi;p__Basidiomycota;c__Tremellomycetes;o__Tremellales;f__;g__;s__
OTU_175	k__Fungi;p__Basidiomycota;c__Tremellomycetes;o__Tremellales;f__Rhynchogastremataceae;g__Papiliotrema;s_

OTU_176	k__Fungi;p__Ascomycota;c__Eurotiomycetes;o__Eurotiales;f__Aspergillaceae;g__Penicillium;s_
OTU_177	k__Fungi;p__;c__;o__;f__;g__;s__
OTU_178	k__Fungi;p__Ascomycota;c__Saccharomycetes;o__Saccharomycetales;f__Saccharomycetales_fam_Incertae_sedis;g__Candida;s__
OTU_179	k__Fungi;p__;c__;o__;f__;g__;s__
OTU_180	No blast hit
OTU_181	k__Fungi;p__;c__;o__;f__;g__;s__
OTU_182	k__Fungi;p__;c__;o__;f__;g__;s__
OTU_183	k__Fungi;p__;c__;o__;f__;g__;s__
OTU_184	k__Fungi;p__;c__;o__;f__;g__;s__
OTU_185	k__Fungi;p__;c__;o__;f__;g__;s__
OTU_186	k__Fungi;p__;c__;o__;f__;g__;s__
OTU_187	k__Fungi;p__Ascomycota;c__Saccharomycetes;o__Saccharomycetales;f__Trichomonascaceae;g__Zygoascus;s_
OTU_188	k__Fungi;p__Basidiomycota;c__Tremellomycetes;o__Tremellales;f__Cuniculitremaceae;g__Fellomyces;s__
OTU_189	k__Fungi;p__Mucoromycota;c__Umbelopsidomycetes;o__Umbelopsidales;f__Umbelopsidaceae;g__Umbelopsis;s__
OTU_190	k__Fungi;p__;c__;o__;f__;g__;s__
OTU_191	k__Fungi;p__Ascomycota;c__Saccharomycetes;o__Saccharomycetales;f__Saccharomycetales_fam_Incertae_sedis;g__Candida;s__
OTU_192	k__Fungi;p__Ascomycota;c__Sordariomycetes;o__Xylariales;f__Xylariaceae;g__Hypoxylon;s__
OTU_193	k__Fungi;p__Ascomycota;c__Sordariomycetes;o__Microascales;f__Microascaceae;g__Microascus;s_
OTU_194	k__Fungi;p__;c__;o__;f__;g__;s__
OTU_195	k__Fungi;p__Ascomycota;c__Saccharomycetes;o__Saccharomycetales;f__Debaryomycetaceae;g__Meyerozyma;s__
OTU_196	k__Fungi;p__Ascomycota;c__Saccharomycetes;o__Saccharomycetales;f__Phaffomycetaceae;g__Barnettozyma;s__Barnettozyma_californica
OTU_197	k__Fungi;p__Ascomycota;c__Saccharomycetes;o__Saccharomycetales;f__Saccharomycetales_fam_Incertae_sedis;g__Candida;s__
OTU_198	k__Fungi;p__Ascomycota;c__Saccharomycetes;o__Saccharomycetales;f__Dipodascaceae;g__Dipodascus;s__
OTU_199	k__Fungi;p__Ascomycota;c__Sordariomycetes;o__Hypocreales;f__Nectriaceae;g__Sarcopodium;s__
OTU_200	k__Fungi;p__Ascomycota;c__Eurotiomycetes;o__Eurotiales;f__Aspergillaceae;g__Penicillium;s__
OTU_201	k__Fungi;p__Ascomycota;c__Eurotiomycetes;o__Chaetothyriales;f__Herpotrichiellaceae;g__Exophiala;s__Exophiala_oligosperma
OTU_202	k__Fungi;p__Ascomycota;c__Eurotiomycetes;o__Eurotiales;f__Aspergillaceae;g__Penicillium;s__
OTU_203	k__Fungi;p__;c__;o__;f__;g__;s__
OTU_204	k__Fungi;p__;c__;o__;f__;g__;s__
OTU_205	k__Fungi;p__;c__;o__;f__;g__;s__
OTU_206	k__Fungi;p__Basidiomycota;c__Cystobasidiomycetes;o__;f__;g__;s__
OTU_207	k__Fungi;p__;c__;o__;f__;g__;s__
OTU_208	k__Fungi;p__Ascomycota;c__Eurotiomycetes;o__Eurotiales;f__Aspergillaceae;g__Aspergillus;s__
OTU_209	k__Fungi;p__Ascomycota;c__Dothideomycetes;o__Capnodiales;f__Cladosporiaceae;g__Cladosporium;s__
OTU_210	k__Fungi;p__;c__;o__;f__;g__;s__
OTU_211	k__Fungi;p__Ascomycota;c__Saccharomycetes;o__Saccharomycetales;f__Dipodascaceae;g__Dipodascus;s__
OTU_212	k__Fungi;p__Ascomycota;c__;o__;f__;g__;s__
OTU_213	k__Fungi;p__;c__;o__;f__;g__;s__
OTU_214	k__Fungi;p__;c__;o__;f__;g__;s__
OTU_215	k__Fungi;p__;c__;o__;f__;g__;s__
OTU_216	k__Fungi;p__Ascomycota;c__Leotiomycetes;o__Helotiales;f__;g__;s__
OTU_217	k__Fungi;p__Basidiomycota;c__Tremellomycetes;o__Tremellales;f__Rhynchogastremataceae;g__Papiliotrema;s__
OTU_218	k__Fungi;p__Ascomycota;c__Eurotiomycetes;o__Eurotiales;f__Aspergillaceae;g__Penicillium;s_
OTU_219	k__Fungi;p__;c__;o__;f__;g__;s__
OTU_220	k__Fungi;p__Basidiomycota;c__Agaricomycetes;o__Agaricales;f__Schizophyllaceae;g__Schizophyllum;s__Schizophyllum_commune
OTU_221	k__Fungi;p__;c__;o__;f__;g__;s__
OTU_222	k__Fungi;p__;c__;o__;f__;g__;s__
OTU_223	k__Fungi;p__Basidiomycota;c__Microbotryomycetes;o__Sporidiobolales;f__Sporidiobolaceae;g__Rhodosporidiobolus;s__Rhodosporidiobolus_ruineniae
OTU_224	k__Fungi;p__;c__;o__;f__;g__;s__
OTU_225	k__Fungi;p__;c__;o__;f__;g__;s__

OTU_226	k__Fungi;p__Ascomycota;c__Dothideomycetes;o__;f__;g__;s__
OTU_227	k__Fungi;p__;c__;o__;f__;g__;s__
OTU_228	k__Fungi;p__;c__;o__;f__;g__;s__
OTU_229	k__Fungi;p__Ascomycota;c__Dothideomycetes;o__Pleosporales;f__Phaeosphaeriaceae;g__Ophiosphaerella;s__
OTU_230	k__Fungi;p__;c__;o__;f__;g__;s__
OTU_231	k__Fungi;p__Ascomycota;c__Dothideomycetes;o__Capnodiales;f__Mycosphaerellaceae;g__Pallidocercospora;s__
OTU_232	k__Fungi;p__;c__;o__;f__;g__;s__
OTU_233	k__Fungi;p__Ascomycota;c__Eurotiomycetes;o__Eurotiales;f__Aspergillaceae;g__Penicillium;s__
OTU_234	k__Fungi;p__;c__;o__;f__;g__;s__
OTU_235	k__Fungi;p__;c__;o__;f__;g__;s__
OTU_236	k__Fungi;p__Ascomycota;c__Eurotiomycetes;o__Eurotiales;f__Aspergillaceae;g__Penicillium;s_
OTU_237	k__Fungi;p__Basidiomycota;c__Tremellomycetes;o__Tremellales;f__Bulleribasidiaceae;g__Hannaella;s__
OTU_238	k__Fungi;p__Ascomycota;c__Saccharomycetes;o__Saccharomycetales;f__Dipodascaceae;g__Dipodascus;s__
OTU_239	k__Fungi;p__;c__;o__;f__;g__;s__
OTU_240	k__Fungi;p__Ascomycota;c__Saccharomycetes;o__Saccharomycetales;f__Debaryomycetaceae;g__Meyerozyma;s__
OTU_241	k__Fungi;p__Ascomycota;c__Dothideomycetes;o__Botryosphaeriales;f__Botryosphaeriaceae;g__Lasiodiplodia;s__
OTU_242	k__Fungi;p__;c__;o__;f__;g__;s__
OTU_243	k__Fungi;p__Ascomycota;c__Sordariomycetes;o__Ophiostomatales;f__Ophiostomataceae;g__Sporothrix;s__
OTU_244	k__Fungi;p__Basidiomycota;c__Exobasidiomycetes;o__Exobasidiales;f__Brachybasidiaceae;g__Meira;s__
OTU_245	k__Fungi;p__;c__;o__;f__;g__;s__
OTU_246	k__Fungi;p__;c__;o__;f__;g__;s__
OTU_247	k__Fungi;p__Ascomycota;c__Eurotiomycetes;o__Chaetothyriales;f__Herpotrichiellaceae;g__Cladophialophora;s__Cladophialophora_boppii
OTU_248	k__Fungi;p__Ascomycota;c__Eurotiomycetes;o__Eurotiales;f__Aspergillaceae;g__Penicillium;s__
OTU_249	k__Fungi;p__Ascomycota;c__Sordariomycetes;o__Xylariales;f__;g__;s__
OTU_250	k__Fungi;p__Ascomycota;c__Lecanoromycetes;o__Lecanorales;f__Stereocaulaceae;g__Lepraria;s__
OTU_251	k__Fungi;p__Basidiomycota;c__Tremellomycetes;o__Tremellales;f__Rhynchogastremataceae;g__Papiliotrema;s__
OTU_252	k__Fungi;p__Basidiomycota;c__Tremellomycetes;o__Trichosporonales;f__Trichosporonaceae;g__Trichosporon;s__
OTU_253	k__Fungi;p__;c__;o__;f__;g__;s__
OTU_254	k__Fungi;p__;c__;o__;f__;g__;s__
OTU_255	k__Fungi;p__Ascomycota;c__Sordariomycetes;o__Sordariales;f__Chaetomiaceae;g__Humicola;s__
OTU_256	k__Fungi;p__;c__;o__;f__;g__;s__
OTU_257	k__Fungi;p__Ascomycota;c__Saccharomycetes;o__Saccharomycetales;f__Metschnikowiaceae;g__Kodamaea;s__Kodamaea_ohmeri
OTU_258	k__Fungi;p__;c__;o__;f__;g__;s__
OTU_259	k__Fungi;p__Ascomycota;c__Eurotiomycetes;o__Eurotiales;f__Trichocomaceae;g__Talaromyces;s__
OTU_260	k__Fungi;p__;c__;o__;f__;g__;s__
OTU_261	k__Fungi;p__Ascomycota;c__;o__;f__;g__;s__
OTU_262	k__Fungi;p__;c__;o__;f__;g__;s__
OTU_263	k__Fungi;p__Basidiomycota;c__Agaricomycetes;o__Agaricales;f__Amanitaceae;g__Amanita;s__
OTU_264	k__Fungi;p__Ascomycota;c__Sordariomycetes;o__Diaporthales;f__;g__;s__
OTU_265	k__Fungi;p__Ascomycota;c__Eurotiomycetes;o__Eurotiales;f__Trichocomaceae;g__Talaromyces;s__
OTU_266	k__Fungi;p__Ascomycota;c__Saccharomycetes;o__Saccharomycetales;f__Debaryomycetaceae;g__Meyerozyma;s__
OTU_267	k__Fungi;p__Rozellomycota;c__;o__;f__;g__;s__
OTU_268	k__Fungi;p__Ascomycota;c__Leotiomycetes;o__Helotiales;f__Helotiales_fam_Incertae_sedis;g__Leptodontidium;s__
OTU_269	k__Fungi;p__Ascomycota;c__Eurotiomycetes;o__Eurotiales;f__Aspergillaceae;g__Aspergillus;s__
OTU_270	k__Fungi;p__;c__;o__;f__;g__;s__
OTU_271	k__Fungi;p__Ascomycota;c__Saccharomycetes;o__Saccharomycetales;f__;g__;s__
OTU_272	k__Fungi;p__Ascomycota;c__Pezizomycetes;o__Pezizales;f__Pyronemataceae;g__Tricharina;s__
OTU_273	k__Fungi;p__Basidiomycota;c__Tremellomycetes;o__Tremellales;f__Bulleribasidiaceae;g__Hannaella;s__
OTU_274	k__Fungi;p__Ascomycota;c__Saccharomycetes;o__Saccharomycetales;f__Dipodascaceae;g__;s__
OTU_275	k__Fungi;p__Ascomycota;c__Sordariomycetes;o__Xylariales;f__;g__;s__
OTU_276	k__Fungi;p__;c__;o__;f__;g__;s__
OTU_277	k__Fungi;p__;c__;o__;f__;g__;s__
OTU_278	k__Fungi;p__;c__;o__;f__;g__;s__
OTU_279	k__Fungi;p__;c__;o__;f__;g__;s__
OTU_280	k__Fungi;p__Basidiomycota;c__Agaricomycetes;o__Boletales;f__Suillaceae;g__Suillus;s__Suillus_placidus

OTU_281	k__Fungi;p__Basidiomycota;c__Microbotryomycetes;o__Sporidiobolales;f__Sporidiobolaceae;g__Rhodotorula;s__
OTU_282	k__Fungi;p__Ascomycota;c__Saccharomycetes;o__Saccharomycetales;f__Pichiaceae;g__Pichia;s__
OTU_283	k__Fungi;p__Basidiomycota;c__Agaricomycetes;o__Cantharellales;f__Hydnaceae;g__;s__
OTU_284	k__Fungi;p__Ascomycota;c__Dothideomycetes;o__Pleosporales;f__Pleosporales_fam_Incertae_sedis;g__Trichobotrys;s__
OTU_285	k__Fungi;p__Ascomycota;c__Sordariomycetes;o__Microascales;f__;g__;s__
OTU_286	k__Fungi;p__;c__;o__;f__;g__;s__
OTU_287	k__Fungi;p__Basidiomycota;c__Tremellomycetes;o__Tremellales;f__Trimorphomycetaceae;g__Saitozyma;s__Saitozyma_podzolica
OTU_288	k__Fungi;p__;c__;o__;f__;g__;s__
OTU_289	k__Fungi;p__Ascomycota;c__Saccharomycetes;o__Saccharomycetales;f__Saccharomycetales_fam_Incertae_sedis;g__Candida;s__Candida_quercitrusa
OTU_290	k__Fungi;p__;c__;o__;f__;g__;s__
OTU_291	k__Fungi;p__Ascomycota;c__Eurotiomycetes;o__Chaetothyriales;f__Cyphellophoraceae;g__Cyphellophora;s__
OTU_292	k__Fungi;p__Basidiomycota;c__Agaricomycetes;o__Polyporales;f__Coriolaceae;g__Trametes;s__
OTU_293	k__Fungi;p__Ascomycota;c__Saccharomycetes;o__Saccharomycetales;f__Saccharomycetales_fam_Incertae_sedis;g__Candida;s__
OTU_294	k__Fungi;p__;c__;o__;f__;g__;s__
OTU_295	k__Fungi;p__;c__;o__;f__;g__;s__
OTU_296	k__Fungi;p__Ascomycota;c__Saccharomycetes;o__Saccharomycetales;f__Dipodascaceae;g__Dipodascus;s_
OTU_297	k__Fungi;p__Ascomycota;c__Eurotiomycetes;o__Eurotiales;f__Trichocomaceae;g__Talaromyces;s__
OTU_298	k__Fungi;p__;c__;o__;f__;g__;s__
OTU_299	k__Fungi;p__;c__;o__;f__;g__;s__
OTU_300	k__Fungi;p__;c__;o__;f__;g__;s__
OTU_301	k__Fungi;p__Ascomycota;c__Sordariomycetes;o__Glomerellales;f__Plectosphaerellaceae;g__Gibellulopsis;s__
OTU_302	k__Fungi;p__Ascomycota;c__Eurotiomycetes;o__Eurotiales;f__Aspergillaceae;g__Penicillium;s__
OTU_303	k__Fungi;p__Ascomycota;c__Eurotiomycetes;o__Eurotiales;f__Aspergillaceae;g__Penicillium;s__
OTU_304	k__Fungi;p__Ascomycota;c__Dothideomycetes;o__Pleosporales;f__;g__;s__
OTU_305	k__Fungi;p__;c__;o__;f__;g__;s__
OTU_306	k__Fungi;p__;c__;o__;f__;g__;s__
OTU_307	k__Fungi;p__;c__;o__;f__;g__;s__
OTU_308	k__Fungi;p__Ascomycota;c__Dothideomycetes;o__Pleosporales;f__Pleosporaceae;g__Bipolaris;s__
OTU_309	No blast hit
OTU_310	k__Fungi;p__;c__;o__;f__;g__;s__
OTU_311	k__Fungi;p__;c__;o__;f__;g__;s__
OTU_312	k__Fungi;p__;c__;o__;f__;g__;s__
OTU_313	k__Fungi;p__;c__;o__;f__;g__;s__
OTU_314	k__Fungi;p__Ascomycota;c__Saccharomycetes;o__Saccharomycetales;f__Phaffomycetaceae;g__Cyberlindnera;s__
OTU_315	k__Fungi;p__Ascomycota;c__Sordariomycetes;o__Xylariales;f__Bartaliniaceae;g__Bartalinia;s__
OTU_316	k__Fungi;p__Ascomycota;c__Dothideomycetes;o__Pleosporales;f__;g__;s__
OTU_317	k__Fungi;p__Basidiomycota;c__Tremellomycetes;o__Tremellales;f__Rhynchogastremataceae;g__Papiliotrema;s_
OTU_318	k__Fungi;p__;c__;o__;f__;g__;s__
OTU_319	k__Fungi;p__Glomeromycota;c__Glomeromycetes;o__Glomerales;f__Glomeraceae;g__Dominikia;s__
OTU_320	k__Fungi;p__;c__;o__;f__;g__;s__
OTU_321	k__Fungi;p__Ascomycota;c__Saccharomycetes;o__Saccharomycetales;f__Metschnikowiaceae;g__Clavispora;s__
OTU_322	k__Fungi;p__Ascomycota;c__Saccharomycetes;o__Saccharomycetales;f__Debaryomycetaceae;g__Meyerozyma;s__
OTU_323	k__Fungi;p__Ascomycota;c__Pezizomycetes;o__Pezizales;f__Pezizaceae;g__;s__
OTU_324	k__Fungi;p__;c__;o__;f__;g__;s__
OTU_325	k__Fungi;p__Ascomycota;c__Saccharomycetes;o__Saccharomycetales;f__Dipodascaceae;g__Dipodascus;s__
OTU_326	k__Fungi;p__Ascomycota;c__Eurotiomycetes;o__Eurotiales;f__Aspergillaceae;g__Penicillium;s__
OTU_327	k__Fungi;p__;c__;o__;f__;g__;s__
OTU_328	k__Fungi;p__Basidiomycota;c__Agaricomycetes;o__Cantharellales;f__Clavulinaceae;g__Clavulina;s__
OTU_329	k__Fungi;p__Ascomycota;c__Sordariomycetes;o__Hypocreales;f__Nectriaceae;g__Neonectria;s__
OTU_330	k__Fungi;p__Ascomycota;c__Saccharomycetes;o__Saccharomycetales;f__Dipodascaceae;g__;s__
OTU_331	k__Fungi;p__Ascomycota;c__Sordariomycetes;o__Hypocreales;f__;g__;s__
OTU_332	k__Fungi;p__Ascomycota;c__Saccharomycetes;o__Saccharomycetales;f__Dipodascaceae;g__Dipodascus;s__
OTU_333	k__Fungi;p__;c__;o__;f__;g__;s__

OTU_334	k__Fungi;p__;c__;o__;f__;g__;s__
OTU_335	k__Fungi;p__Ascomycota;c__Saccharomycetes;o__Saccharomycetales;f__Dipodascaceae;g__Dipodascus;s__
OTU_336	k__Fungi;p__;c__;o__;f__;g__;s__
OTU_337	k__Fungi;p__Ascomycota;c__Saccharomycetes;o__Saccharomycetales;f__Dipodascaceae;g__Dipodascus;s__
OTU_338	k__Fungi;p__;c__;o__;f__;g__;s__
OTU_339	k__Fungi;p__;c__;o__;f__;g__;s__
OTU_340	k__Fungi;p__Basidiomycota;c__Tremellomycetes;o__Tremellales;f__Rhynchogastremataceae;g__Papiliotrema;s__
OTU_341	k__Fungi;p__;c__;o__;f__;g__;s__
OTU_342	k__Fungi;p__Ascomycota;c__Archaeorhizomycetes;o__;f__;g__;s__
OTU_343	k__Fungi;p__;c__;o__;f__;g__;s__
OTU_344	k__Fungi;p__;c__;o__;f__;g__;s__
OTU_345	k__Fungi;p__Ascomycota;c__;o__;f__;g__;s__
OTU_346	k__Fungi;p__Ascomycota;c__Dothideomycetes;o__Capnodiales;f__Cladosporiaceae;g__;s__
OTU_347	k__Fungi;p__Ascomycota;c__Sordariomycetes;o__Hypocreales;f__Nectriaceae;g__Fusarium;s__
OTU_348	k__Fungi;p__Ascomycota;c__;o__;f__;g__;s__
OTU_349	No blast hit
OTU_350	k__Fungi;p__Basidiomycota;c__Tremellomycetes;o__Tremellales;f__Rhynchogastremataceae;g__Papiliotrema;s__
OTU_351	k__Fungi;p__;c__;o__;f__;g__;s__
OTU_352	k__Fungi;p__Ascomycota;c__Saccharomycetes;o__Saccharomycetales;f__Dipodascaceae;g__Dipodascus;s__
OTU_353	k__Fungi;p__;c__;o__;f__;g__;s__
OTU_354	k__Fungi;p__Basidiomycota;c__Tremellomycetes;o__Tremellales;f__Trimorphomycetaceae;g__;s__
OTU_355	k__Fungi;p__;c__;o__;f__;g__;s__
OTU_356	k__Fungi;p__Ascomycota;c__Eurotiomycetes;o__Eurotiales;f__Aspergillaceae;g__Penicillium;s_
OTU_357	k__Fungi;p__;c__;o__;f__;g__;s__
OTU_358	k__Fungi;p__Ascomycota;c__Saccharomycetes;o__Saccharomycetales;f__Metschnikowiaceae;g__Metschnikowia;s__
OTU_359	k__Fungi;p__Ascomycota;c__Eurotiomycetes;o__Eurotiales;f__Aspergillaceae;g__Penicillium;s__
OTU_360	k__Fungi;p__;c__;o__;f__;g__;s__
OTU_361	k__Fungi;p__Ascomycota;c__Sordariomycetes;o__Sordariales;f__Lasiosphaeriaceae;g__Fimetariella;s__
OTU_362	k__Fungi;p__;c__;o__;f__;g__;s__
OTU_363	k__Fungi;p__;c__;o__;f__;g__;s__
OTU_364	k__Fungi;p__;c__;o__;f__;g__;s__
OTU_365	k__Fungi;p__Chytridiomycota;c__Rhizophydiomycetes;o__Rhizophydiales;f__Terramycetaceae;g__Boothiomyces;s__
OTU_366	k__Fungi;p__;c__;o__;f__;g__;s__
OTU_367	k__Fungi;p__Ascomycota;c__Eurotiomycetes;o__Eurotiales;f__Aspergillaceae;g__Penicillium;s__
OTU_368	k__Fungi;p__;c__;o__;f__;g__;s__
OTU_369	k__Fungi;p__Ascomycota;c__Dothideomycetes;o__Capnodiales;f__Cladosporiaceae;g__;s__
OTU_370	k__Fungi;p__Ascomycota;c__Eurotiomycetes;o__Eurotiales;f__Aspergillaceae;g__Penicillium;s__
OTU_371	k__Fungi;p__;c__;o__;f__;g__;s__
OTU_372	k__Fungi;p__;c__;o__;f__;g__;s__
OTU_373	k__Fungi;p__;c__;o__;f__;g__;s__
OTU_374	k__Fungi;p__;c__;o__;f__;g__;s__
OTU_375	k__Fungi;p__;c__;o__;f__;g__;s__
OTU_376	k__Fungi;p__Ascomycota;c__Eurotiomycetes;o__Chaetothyriales;f__Herpotrichiellaceae;g__Exophiala;s__
OTU_377	k__Fungi;p__Basidiomycota;c__Agaricomycetes;o__Agaricales;f__Agaricales_fam_Incertae_sedis;g__Digitatispora;s__
OTU_378	k__Fungi;p__;c__;o__;f__;g__;s__
OTU_379	k__Fungi;p__;c__;o__;f__;g__;s__
OTU_380	k__Fungi;p__Ascomycota;c__Eurotiomycetes;o__Eurotiales;f__Aspergillaceae;g__Aspergillus;s__
OTU_381	k__Fungi;p__Ascomycota;c__Saccharomycetes;o__Saccharomycetales;f__Pichiaceae;g__Pichia;s__Pichia_klvyveri
OTU_382	k__Fungi;p__Ascomycota;c__Dothideomycetes;o__Pleosporales;f__Didymosphaeriaceae;g__Montagnula;s__
OTU_383	k__Fungi;p__Basidiomycota;c__Tremellomycetes;o__Tremellales;f__Cuniculitremaceae;g__Fellomyces;s__
OTU_384	k__Fungi;p__;c__;o__;f__;g__;s__
OTU_385	k__Fungi;p__Ascomycota;c__Saccharomycetes;o__Saccharomycetales;f__Saccharomycetales_fam_Incertae_sedis;g__Candida;s__
OTU_386	k__Fungi;p__Ascomycota;c__Saccharomycetes;o__Saccharomycetales;f__Debaryomycetaceae;g__Meyerozyma;s__
OTU_387	k__Fungi;p__Basidiomycota;c__Agaricomycetes;o__Atheliales;f__Atheliaceae;g__Piloderma;s__

OTU_388	k__Fungi;p__Basidiomycota;c__Cystobasidiomycetes;o__Cystobasidiales;f__Cystobasidiaceae;g__Cystobasidium;s__
OTU_389	k__Fungi;p__;c__;o__;f__;g__;s__
OTU_390	k__Fungi;p__Basidiomycota;c__Tremellomycetes;o__Tremellales;f__Rhynchogastremataceae;g__Papiliotrema;s__
OTU_391	k__Fungi;p__;c__;o__;f__;g__;s__
OTU_392	k__Fungi;p__Ascomycota;c__Sordariomycetes;o__Hypocreales;f__Cordycipitaceae;g__Beauveria;s__
OTU_393	k__Fungi;p__Glomeromycota;c__Glomeromycetes;o__Glomerales;f__Glomeraceae;g__;s__
OTU_394	k__Fungi;p__;c__;o__;f__;g__;s__
OTU_395	k__Fungi;p__;c__;o__;f__;g__;s__
OTU_396	k__Fungi;p__;c__;o__;f__;g__;s__
OTU_397	k__Fungi;p__Basidiomycota;c__Tremellomycetes;o__Tremellales;f__Rhynchogastremataceae;g__Papiliotrema;s__
OTU_398	k__Fungi;p__Ascomycota;c__Eurotiomycetes;o__Chaetothyriales;f__Cyphellophoraceae;g__Cyphellophora;s__
OTU_399	k__Fungi;p__;c__;o__;f__;g__;s__
OTU_400	k__Fungi;p__;c__;o__;f__;g__;s__
OTU_401	k__Fungi;p__;c__;o__;f__;g__;s__
OTU_402	k__Fungi;p__Basidiomycota;c__;o__;f__;g__;s__
OTU_403	k__Fungi;p__Basidiomycota;c__Wallemiomycetes;o__Wallemiales;f__Wallemiaceae;g__Wallemia;s__
OTU_404	k__Fungi;p__;c__;o__;f__;g__;s__
OTU_405	k__Fungi;p__Basidiomycota;c__Agaricomycetes;o__Agaricales;f__Lyophyllaceae;g__Lyophyllum;s__
OTU_406	k__Fungi;p__;c__;o__;f__;g__;s__
OTU_407	k__Fungi;p__;c__;o__;f__;g__;s__
OTU_408	k__Fungi;p__Ascomycota;c__Saccharomycetes;o__Saccharomycetales;f__Debaryomycetaceae;g__Meyerozyma;s__
OTU_409	k__Fungi;p__;c__;o__;f__;g__;s__
OTU_410	k__Fungi;p__;c__;o__;f__;g__;s__
OTU_411	k__Fungi;p__;c__;o__;f__;g__;s__
OTU_412	k__Fungi;p__;c__;o__;f__;g__;s__
OTU_413	k__Fungi;p__Basidiomycota;c__Agaricomycetes;o__Cantharellales;f__Clavulinaceae;g__;s__
OTU_414	k__Fungi;p__Ascomycota;c__Saccharomycetes;o__Saccharomycetales;f__Debaryomycetaceae;g__Meyerozyma;s__
OTU_415	k__Fungi;p__;c__;o__;f__;g__;s__
OTU_416	k__Fungi;p__;c__;o__;f__;g__;s__
OTU_417	k__Fungi;p__;c__;o__;f__;g__;s__
OTU_418	k__Fungi;p__Ascomycota;c__Eurotiomycetes;o__Eurotiales;f__Aspergillaceae;g__Penicillium;s__
OTU_419	k__Fungi;p__;c__;o__;f__;g__;s__
OTU_420	k__Fungi;p__;c__;o__;f__;g__;s__
OTU_421	k__Fungi;p__;c__;o__;f__;g__;s__
OTU_422	k__Fungi;p__Basidiomycota;c__Tremellomycetes;o__Tremellales;f__;g__;s__
OTU_423	k__Fungi;p__Ascomycota;c__Saccharomycetes;o__Saccharomycetales;f__Dipodascaceae;g__;s__
OTU_424	k__Fungi;p__Ascomycota;c__Eurotiomycetes;o__Eurotiales;f__Trichocomaceae;g__Talaromyces;s__
OTU_425	k__Fungi;p__Ascomycota;c__Eurotiomycetes;o__Eurotiales;f__Aspergillaceae;g__Penicillium;s__
OTU_426	k__Fungi;p__;c__;o__;f__;g__;s__
OTU_427	k__Fungi;p__Ascomycota;c__Dothideomycetes;o__Mytilinidales;f__Gloniaceae;g__Cenococcum;s__
OTU_428	k__Fungi;p__;c__;o__;f__;g__;s__
OTU_429	k__Fungi;p__;c__;o__;f__;g__;s__
OTU_430	k__Fungi;p__Ascomycota;c__Saccharomycetes;o__Saccharomycetales;f__Saccharomycetales_fam_Incertae_sedis;g__ Candida;s__
OTU_431	k__Fungi;p__Ascomycota;c__Dothideomycetes;o__Pleosporales;f__;g__;s__
OTU_432	k__Fungi;p__Ascomycota;c__Eurotiomycetes;o__Eurotiales;f__Aspergillaceae;g__Penicillium;s__
OTU_433	k__Fungi;p__Ascomycota;c__Eurotiomycetes;o__Eurotiales;f__Aspergillaceae;g__Penicillium;s__
OTU_434	k__Fungi;p__Ascomycota;c__Saccharomycetes;o__Saccharomycetales;f__Pichiaceae;g__Pichia;s__Pichia_fermentans
OTU_435	k__Fungi;p__;c__;o__;f__;g__;s__
OTU_436	k__Fungi;p__;c__;o__;f__;g__;s__
OTU_437	k__Fungi;p__Basidiomycota;c__Tremellomycetes;o__Tremellales;f__;g__;s__
OTU_438	k__Fungi;p__;c__;o__;f__;g__;s__
OTU_439	k__Fungi;p__Ascomycota;c__Saccharomycetes;o__Saccharomycetales;f__Dipodascaceae;g__Dipodascus;s__
OTU_440	k__Fungi;p__;c__;o__;f__;g__;s__
OTU_441	k__Fungi;p__Basidiomycota;c__Agaricomycetes;o__Gomphales;f__Gomphaceae;g__Ramaria;s__
OTU_442	k__Fungi;p__Ascomycota;c__Dothideomycetes;o__Dothideales;f__Aureobasidiaceae;g__Aureobasidium;s__
OTU_443	k__Fungi;p__;c__;o__;f__;g__;s__

OTU_444	k__Fungi;p__;c__;o__;f__;g__;s__
OTU_445	k__Fungi;p__Basidiomycota;c__Agaricomycetes;o__Agaricales;f__Amanitaceae;g__Amanita;s__
OTU_446	k__Fungi;p__Mortierellomycota;c__Mortierellomycetes;o__Mortierellales;f__Mortierellaceae;g__;s__
OTU_447	k__Fungi;p__Basidiomycota;c__Agaricomycetes;o__Agaricales;f__Pleurotaceae;g__Pleurotus;s__
OTU_448	k__Fungi;p__;c__;o__;f__;g__;s__
OTU_449	k__Fungi;p__Ascomycota;c__Saccharomycetes;o__Saccharomycetales;f__Dipodascaceae;g__Dipodascus;s__
OTU_450	k__Fungi;p__Ascomycota;c__Eurotiomycetes;o__Eurotiales;f__Aspergillaceae;g__Penicillium;s__
OTU_451	k__Fungi;p__;c__;o__;f__;g__;s__
OTU_452	k__Fungi;p__;c__;o__;f__;g__;s__
OTU_453	k__Fungi;p__Ascomycota;c__Saccharomycetes;o__Saccharomycetales;f__Dipodascaceae;g__Dipodascus;s__
OTU_454	No blast hit
OTU_455	k__Fungi;p__Ascomycota;c__Saccharomycetes;o__Saccharomycetales;f__Dipodascaceae;g__Dipodascus;s__
OTU_456	k__Fungi;p__Ascomycota;c__Eurotiomycetes;o__Eurotiales;f__Aspergillaceae;g__Aspergillus;s__
OTU_457	k__Fungi;p__;c__;o__;f__;g__;s__
OTU_458	k__Fungi;p__;c__;o__;f__;g__;s__
OTU_459	k__Fungi;p__;c__;o__;f__;g__;s__
OTU_460	k__Fungi;p__;c__;o__;f__;g__;s__
OTU_461	k__Fungi;p__Ascomycota;c__Eurotiomycetes;o__Eurotiales;f__Aspergillaceae;g__Penicillium;s__
OTU_462	k__Fungi;p__Basidiomycota;c__Agaricomycetes;o__Agaricales;f__Tricholomataceae;g__Tricholoma;s__Tricholoma_ portentosum
OTU_463	k__Fungi;p__;c__;o__;f__;g__;s__
OTU_464	k__Fungi;p__Ascomycota;c__Saccharomycetes;o__Saccharomycetales;f__Debaryomycetaceae;g__Meyerozyma;s__
OTU_465	k__Fungi;p__Ascomycota;c__Saccharomycetes;o__Saccharomycetales;f__Debaryomycetaceae;g__Meyerozyma;s__
OTU_466	k__Fungi;p__Ascomycota;c__Eurotiomycetes;o__Eurotiales;f__Aspergillaceae;g__Penicillium;s__
OTU_467	k__Fungi;p__;c__;o__;f__;g__;s__
OTU_468	k__Fungi;p__;c__;o__;f__;g__;s__
OTU_469	k__Fungi;p__Ascomycota;c__Dothideomycetes;o__Capnodiales;f__Mycosphaerellaceae;g__Zasmidium;s__
OTU_470	k__Fungi;p__;c__;o__;f__;g__;s__
OTU_471	k__Fungi;p__Basidiomycota;c__Agaricomycetes;o__Agaricales;f__Amanitaceae;g__Amanita;s__
OTU_472	k__Fungi;p__Basidiomycota;c__Microbotryomycetes;o__Sporidiobolales;f__Sporidiobolaceae;g__Rhodosporidiobolu s;s__Rhodosporidiobolus_odoratus
OTU_473	k__Fungi;p__;c__;o__;f__;g__;s__
OTU_474	k__Fungi;p__Ascomycota;c__Eurotiomycetes;o__Eurotiales;f__Trichocomaceae;g__Talaromyces;s__
OTU_475	k__Fungi;p__Ascomycota;c__Sordariomycetes;o__Hypocreales;f__Nectriaceae;g__Stephanonectria;s__Stephanonectr ia_keithii
OTU_476	k__Fungi;p__;c__;o__;f__;g__;s__
OTU_477	k__Fungi;p__Ascomycota;c__Saccharomycetes;o__Saccharomycetales;f__Dipodascaceae;g__Dipodascus;s__
OTU_478	k__Fungi;p__;c__;o__;f__;g__;s__
OTU_479	k__Fungi;p__;c__;o__;f__;g__;s__
OTU_480	k__Fungi;p__Ascomycota;c__Saccharomycetes;o__Saccharomycetales;f__Debaryomycetaceae;g__Meyerozyma;s__
OTU_481	k__Fungi;p__Ascomycota;c__Leotiomycetes;o__Thelebolales;f__Pseudeurotiaceae;g__Pseudogymnoascus;s__Pseudo gymnoascus_roseus
OTU_482	k__Fungi;p__;c__;o__;f__;g__;s__
OTU_483	No blast hit
OTU_484	k__Fungi;p__Ascomycota;c__Dothideomycetes;o__Capnodiales;f__Cladosporiaceae;g__Cladosporium;s__
OTU_485	k__Fungi;p__;c__;o__;f__;g__;s__
OTU_486	k__Fungi;p__;c__;o__;f__;g__;s__
OTU_487	k__Fungi;p__Ascomycota;c__Dothideomycetes;o__Pleosporales;f__Sporormiaceae;g__Preussia;s__
OTU_488	k__Fungi;p__;c__;o__;f__;g__;s__
OTU_489	k__Fungi;p__Ascomycota;c__Eurotiomycetes;o__Eurotiales;f__Elaphomycetaceae;g__Elaphomyces;s__
OTU_490	k__Fungi;p__Ascomycota;c__Eurotiomycetes;o__Eurotiales;f__Aspergillaceae;g__Aspergillus;s__
OTU_491	k__Fungi;p__Ascomycota;c__Saccharomycetes;o__Saccharomycetales;f__Dipodascaceae;g__Dipodascus;s__
OTU_492	k__Fungi;p__Ascomycota;c__Saccharomycetes;o__Saccharomycetales;f__Dipodascaceae;g__Dipodascus;s__
OTU_493	k__Fungi;p__Ascomycota;c__Saccharomycetes;o__Saccharomycetales;f__Saccharomycetales_fam_Incertae_sedis;g__ Candida;s__
OTU_494	k__Fungi;p__Ascomycota;c__Sordariomycetes;o__Hypocreales;f__Nectriaceae;g__Fusicolla;s__
OTU_495	k__Fungi;p__Ascomycota;c__Saccharomycetes;o__Saccharomycetales;f__Debaryomycetaceae;g__Meyerozyma;s__

OTU_496	k__Fungi;p__Basidiomycota;c__Tremellomycetes;o__Tremellales;f__Rhynchogastremataceae;g__Papiliotrema;s__
OTU_497	k__Fungi;p__;c__;o__;f__;g__;s__
OTU_498	k__Fungi;p__;c__;o__;f__;g__;s__
OTU_499	k__Fungi;p__Basidiomycota;c__Wallemiomycetes;o__Wallemiales;f__Wallemiaceae;g__Wallemia;s__
OTU_500	k__Fungi;p__Ascomycota;c__Eurotiomycetes;o__Eurotiales;f__Aspergillaceae;g__Aspergillus;s__
OTU_501	k__Fungi;p__Ascomycota;c__Eurotiomycetes;o__Eurotiales;f__Aspergillaceae;g__Aspergillus;s__
OTU_502	k__Fungi;p__;c__;o__;f__;g__;s__
OTU_503	k__Fungi;p__;c__;o__;f__;g__;s__
OTU_504	k__Fungi;p__;c__;o__;f__;g__;s__
OTU_505	k__Fungi;p__Ascomycota;c__Eurotiomycetes;o__Eurotiales;f__Aspergillaceae;g__Penicillium;s__
OTU_506	k__Fungi;p__;c__;o__;f__;g__;s__
OTU_507	k__Fungi;p__Ascomycota;c__Sordariomycetes;o__Hypocreales;f__Nectriaceae;g__;s__
OTU_508	k__Fungi;p__Basidiomycota;c__Agaricomycetes;o__Atheliales;f__Atheliaceae;g__Piloderma;s__
OTU_509	k__Fungi;p__Ascomycota;c__Saccharomycetes;o__Saccharomycetales;f__Saccharomycodaceae;g__Hanseniaspora;s__
OTU_510	No blast hit
OTU_511	k__Fungi;p__Ascomycota;c__Eurotiomycetes;o__Eurotiales;f__Aspergillaceae;g__Monascus;s__
OTU_512	k__Fungi;p__;c__;o__;f__;g__;s__
OTU_513	k__Fungi;p__Basidiomycota;c__;o__;f__;g__;s__
OTU_514	k__Fungi;p__Ascomycota;c__Saccharomycetes;o__Saccharomycetales;f__Dipodascaceae;g__;s__
OTU_515	k__Fungi;p__Ascomycota;c__Eurotiomycetes;o__Eurotiales;f__Aspergillaceae;g__Penicillium;s__
OTU_516	k__Fungi;p__Ascomycota;c__Saccharomycetes;o__Saccharomycetales;f__Dipodascaceae;g__Dipodascus;s__
OTU_517	k__Fungi;p__;c__;o__;f__;g__;s__
OTU_518	No blast hit
OTU_519	k__Fungi;p__;c__;o__;f__;g__;s__
OTU_520	k__Fungi;p__;c__;o__;f__;g__;s__
OTU_521	k__Fungi;p__Ascomycota;c__Saccharomycetes;o__Saccharomycetales;f__Saccharomycetales_fam_Incertae_sedis;g__Candida;s__
OTU_522	k__Fungi;p__Ascomycota;c__Eurotiomycetes;o__Eurotiales;f__Aspergillaceae;g__Penicillium;s__
OTU_523	k__Fungi;p__Basidiomycota;c__Agaricomycetes;o__Russulales;f__Russulaceae;g__Lactifluus;s__
OTU_524	k__Fungi;p__;c__;o__;f__;g__;s__
OTU_525	k__Fungi;p__Ascomycota;c__Dothideomycetes;o__Capnodiales;f__Mycosphaerellaceae;g__Cercospora;s__
OTU_526	k__Fungi;p__;c__;o__;f__;g__;s__
OTU_527	k__Fungi;p__Ascomycota;c__Sordariomycetes;o__Hypocreales;f__Nectriaceae;g__Fusarium;s__
OTU_528	k__Fungi;p__Ascomycota;c__;o__;f__;g__;s__
OTU_529	k__Fungi;p__;c__;o__;f__;g__;s__
OTU_530	k__Fungi;p__Ascomycota;c__Saccharomycetes;o__Saccharomycetales;f__Dipodascaceae;g__Dipodascus;s__
OTU_531	k__Fungi;p__;c__;o__;f__;g__;s__
OTU_532	k__Fungi;p__Ascomycota;c__Eurotiomycetes;o__Eurotiales;f__Aspergillaceae;g__Penicillium;s__
OTU_533	k__Fungi;p__;c__;o__;f__;g__;s__
OTU_534	k__Fungi;p__Ascomycota;c__Eurotiomycetes;o__Eurotiales;f__Aspergillaceae;g__Aspergillus;s__
OTU_535	k__Fungi;p__;c__;o__;f__;g__;s__
OTU_536	k__Fungi;p__Ascomycota;c__;o__;f__;g__;s__
OTU_537	k__Fungi;p__Ascomycota;c__Saccharomycetes;o__Saccharomycetales;f__Saccharomycetales_fam_Incertae_sedis;g__Candida;s__
OTU_538	k__Fungi;p__;c__;o__;f__;g__;s__
OTU_539	k__Fungi;p__;c__;o__;f__;g__;s__
OTU_540	k__Fungi;p__Ascomycota;c__Dothideomycetes;o__Pleosporales;f__;g__;s__
OTU_541	k__Fungi;p__Ascomycota;c__Eurotiomycetes;o__Eurotiales;f__Aspergillaceae;g__Monascus;s__
OTU_542	k__Fungi;p__;c__;o__;f__;g__;s__
OTU_543	k__Fungi;p__;c__;o__;f__;g__;s__
OTU_544	k__Fungi;p__Ascomycota;c__Dothideomycetes;o__Pleosporales;f__Pleosporales_fam_Incertae_sedis;g__Latorua;s__
OTU_545	k__Fungi;p__;c__;o__;f__;g__;s__
OTU_546	k__Fungi;p__;c__;o__;f__;g__;s__
OTU_547	k__Fungi;p__Ascomycota;c__Saccharomycetes;o__Saccharomycetales;f__Saccharomycetales_fam_Incertae_sedis;g__Candida;s__
OTU_548	k__Fungi;p__;c__;o__;f__;g__;s__

OTU_549	k__Fungi;p__;c__;o__;f__;g__;s__
OTU_550	k__Fungi;p__;c__;o__;f__;g__;s__
OTU_551	k__Fungi;p__Ascomycota;c__Eurotiomycetes;o__Chaetothyriales;f__Cyphellophoraceae;g__Cyphellophora;s__
OTU_552	k__Fungi;p__Basidiomycota;c__Exobasidiomycetes;o__Exobasidiales;f__Brachybasidiaceae;g__Meira;s__
OTU_553	k__Fungi;p__;c__;o__;f__;g__;s__
OTU_554	k__Fungi;p__Ascomycota;c__Saccharomycetes;o__Saccharomycetales;f__Dipodascaceae;g__Dipodascus;s__
OTU_555	k__Fungi;p__Ascomycota;c__Eurotiomycetes;o__Eurotiales;f__Aspergillaceae;g__Monascus;s__
OTU_556	k__Fungi;p__Ascomycota;c__Dothideomycetes;o__Pleosporales;f__;g__;s__
OTU_557	k__Fungi;p__Ascomycota;c__Saccharomycetes;o__Saccharomycetales;f__Dipodascaceae;g__Dipodascus;s__
OTU_558	k__Fungi;p__Basidiomycota;c__Tremellomycetes;o__Tremellales;f__Rhynchogastremataceae;g__Papiliotrema;s__
OTU_559	k__Fungi;p__;c__;o__;f__;g__;s__
OTU_560	k__Fungi;p__Ascomycota;c__Sordariomycetes;o__Sordariales;f__Cephalothecaceae;g__Phialemonium;s__Phialemonium_dimorphosporum
OTU_561	k__Fungi;p__Ascomycota;c__Saccharomycetes;o__Saccharomycetales;f__Dipodascaceae;g__Dipodascus;s__
OTU_562	k__Fungi;p__Ascomycota;c__Dothideomycetes;o__Capnodiales;f__Cladosporiaceae;g__Cladosporium;s__
OTU_563	k__Fungi;p__Basidiomycota;c__Agaricomycetes;o__Agaricales;f__Clavariaceae;g__Clavaria;s__
OTU_564	k__Fungi;p__;c__;o__;f__;g__;s__
OTU_565	k__Fungi;p__;c__;o__;f__;g__;s__
OTU_566	k__Fungi;p__;c__;o__;f__;g__;s__
OTU_567	k__Fungi;p__;c__;o__;f__;g__;s__
OTU_568	k__Fungi;p__Basidiomycota;c__Tremellomycetes;o__Tremellales;f__Rhynchogastremataceae;g__Papiliotrema;s__
OTU_569	k__Fungi;p__;c__;o__;f__;g__;s__
OTU_570	k__Fungi;p__Ascomycota;c__Dothideomycetes;o__Capnodiales;f__Mycosphaerellaceae;g__Mycocentrospora;s__Mycocentrospora_acerina
OTU_571	k__Fungi;p__Rozellomycota;c__;o__;f__;g__;s__
OTU_572	k__Fungi;p__Ascomycota;c__Eurotiomycetes;o__Eurotiales;f__Aspergillaceae;g__Penicillium;s__
OTU_573	k__Fungi;p__Ascomycota;c__Saccharomycetes;o__Saccharomycetales;f__Dipodascaceae;g__;s__
OTU_574	k__Fungi;p__;c__;o__;f__;g__;s__
OTU_575	k__Fungi;p__Ascomycota;c__Sordariomycetes;o__;f__;g__;s__
OTU_576	k__Fungi;p__;c__;o__;f__;g__;s__
OTU_577	k__Fungi;p__Ascomycota;c__Pezizomycetes;o__Pezizales;f__Pyronemataceae;g__Byssonectria;s__
OTU_578	k__Fungi;p__;c__;o__;f__;g__;s__
OTU_579	k__Fungi;p__;c__;o__;f__;g__;s__

Table S2. The species and relative abundance of harmful fungi in each sample.

Sample ID	*Trichoderma atroviride*	*Fusarium equiseti*
MCK1	0.1017%	0.0458%
MCK2	0.1271%	0.0051%
MCK3	0.2695%	0.0000%
GDM1	0.1703%	0.0000%
GDM2	0.1805%	0.0000%
GDM3	0.1703%	0.0000%
GXM1	0.0483%	0.0000%
GXM2	0.1068%	0.0000%
GXM3	0.1017%	0.0000%
ACK1	0.1424%	0.0025%
ACK2	0.0483%	0.0000%
ACK3	0.3381%	0.0025%
HNA1	0.0763%	0.0000%
HNA2	0.0686%	0.0000%
HNA3	0.0381%	0.0000%
GXA1	0.0483%	0.0000%
GXA2	0.0356%	0.0000%
GXA3	0.0508%	0.0000%
